# The combination of FLT3 and SYK kinase inhibitors is toxic to leukaemia cells with CBL mutations

**DOI:** 10.1111/jcmm.14820

**Published:** 2020-01-14

**Authors:** Ellen Weisberg, Chengcheng Meng, Abigail E. Case, Hong L. Tiv, Prafulla C. Gokhale, Anthia A. Toure, Sara Buhrlage, Xiaoxi Liu, Jinhua Wang, Nathanael Gray, Richard Stone, Sophia Adamia, Eric Winer, Martin Sattler, James D. Griffin

**Affiliations:** ^1^ Department of Medical Oncology Dana‐Farber Cancer Institute Boston MA USA; ^2^ Department of Medicine Harvard Medical School Boston MA USA; ^3^ Experimental Therapeutics Core and Belfer Center for Applied Cancer Science Dana‐Farber Cancer Institute Harvard Medical School Boston MA USA; ^4^ Department of Cancer Biology Dana‐Farber Cancer Institute Harvard Medical School Boston MA USA; ^5^ Department of Biological Chemistry and Molecular Pharmacology Harvard Medical School Boston MA USA

**Keywords:** AML, FLT3, leukaemia, mutant CBL, tyrosine kinase

## Abstract

Mutations in the E3 ubiquitin ligase CBL, found in several myeloid neoplasms, lead to decreased ubiquitin ligase activity. In murine systems, these mutations are associated with cytokine‐independent proliferation, thought to result from the activation of hematopoietic growth receptors, including FLT3 and KIT. Using cell lines and primary patient cells, we compared the activity of a panel of FLT3 inhibitors currently being used or tested in AML patients and also evaluated the effects of inhibition of the non‐receptor tyrosine kinase, SYK. We show that FLT3 inhibitors ranging from promiscuous to highly targeted are potent inhibitors of growth of leukaemia cells expressing mutant CBL in vitro*,* and we demonstrate in vivo efficacy of midostaurin using mouse models of mutant CBL. Potentiation of effects of targeted FLT3 inhibition by SYK inhibition has been demonstrated in models of mutant FLT3‐positive AML and AML characterized by hyperactivated SYK. Here, we show that targeted SYK inhibition similarly enhances the effects of midostaurin and other FLT3 inhibitors against mutant CBL‐positive leukaemia. Taken together, our results support the notion that mutant CBL‐expressing myeloid leukaemias are highly sensitive to available FLT3 inhibitors and that this effect can be significantly augmented by optimum inhibition of SYK kinase.

## 1 INTRODUCTION

Blood disorders that fall under the spectrum of myeloid neoplasms include myelodysplastic syndrome (MDS), myeloproliferative neoplasms (MPNs), acute myeloid leukaemia (AML) and MDS/MPN overlap syndromes. AML is a hematopoietic malignancy that is characterized by abnormal myeloid progenitor cell growth and a partial block in cell differentiation.^1^ There are several commonly detected mutations that confer a growth advantage to leukaemic cells, such as mutations in receptor tyrosine kinases (RTKs) (FLT3 and KIT, for example), non‐receptor tyrosine kinases (non‐RTKs) (JAK2 and ABL) and downstream signalling molecules such as RAS. Loss of function or deletion of genes that normally turn off growth signalling may also be oncogenic. One example is the E3 ubiquitin ligase, CBL, which is mutated in approximately 10% of myeloid neoplasms, with a low occurrence of 1.1% in AML/MDS and a higher occurrences of 16% in AMLs carrying inv(16)/t(16;16) chromosomal abnormalities (CBL splicing mutations) or 13%‐15% in patients with juvenile myelomonocytic leukaemia (JMML) or chronic myelomonocytic leukaemia (CMML).^2^, ^3^, ^4^, ^5^, ^6^ CBL mutations cluster in exons 8 and 9, with approximately 85% occurring as point mutations and 15% presenting as small deletions in the linker or RING finger domain of CBL.^2^, ^7^, ^8^ These CBL mutations are generally thought to decrease E3 ubiquitin ligase activity.

Interestingly, CBL is known to have as targets several non‐RTKs, such as SYK, and RTKs including FLT3, KIT and FMS, as well as the oncogenic variants of those RTKs.^9^, ^10^ In myeloid neoplasms, mutations in CBL are thought to decrease turnover of these RTKs and promote growth and viability signalling. In these diseases, CBL, functionally and genetically, acts like a tumour suppressor.^8^ This hypothesis is supported by a number of studies demonstrating spontaneous activation of RTK signalling pathways in cells with CBL mutations and also limited studies where multitargeted tyrosine kinase inhibitors (TKI's) such as midostaurin or SU11248^2^, ^8^, ^9^, ^11^ can reverse factor independence or factor‐hyperresponsiveness of leukaemia cells.^12^

As the direct targeting of CBL is a challenge, finding upstream/downstream vulnerabilities related to mutated CBL is important to develop a successful treatment approach. Previous work has established that the effects of mutated CBL and mutated FLT3 on intracellular signalling, particularly transforming hyperstimulation of signalling molecules downstream of FLT3, are similar,^12^ and we thus sought to comprehensively evaluate clinical‐grade inhibitors of FLT3 for efficacy against mutant CBL AML. In this study, we have compared the activity of multiple TKIs that have a varied spectrum of activity against FLT3 or KIT and which are available for clinical use or testing, including the *N*‐indolocarbazole midostaurin (PKC412; Rydapt; Novartis Pharma AG),^13^, ^14^ the pyrazinecarboxamide derivative, gilteritinib (ASP2215; XOSPATA; Astellas Pharma US, Inc),^15^, ^16^ the highly selective FLT3 inhibitor, quizartinib (AC220; Daiichi Sankyo),^17^, ^18^ crenolanib besylate (CP‐868596; AROG Pharmaceuticals, LLC)^19^ and sorafenib (Nexavar; co‐developed and co‐marketed by Bayer and Onyx Pharmaceuticals).^20^ In our cell line model, co‐expression of wt FLT3, but not c‐KIT, in Ba/F3 cells with mutant CBL was found to be necessary and sufficient to achieve growth factor‐independent growth^12^ and we therefore tested the effects of the midostaurin and other FLT3 inhibitors on these cells.

For mutant FLT3‐positive AML patients to achieve maximum clinical benefit, it has been imperative that midostaurin be administered in combination with other anti‐cancer agents. We and others have previously shown that inhibition of the non‐receptor cytoplasmic tyrosine kinase (non‐RTK) SYK, established to play a critical role in AML transformation, potentiates the anti‐leukaemic activity of targeted FLT3 inhibition in models of FLT3‐ITD AML and AML characterized by hyperactivated SYK.^21^, ^22^ Given the potential of SYK as a putative therapeutic target in AML and the demonstrated ability of additional SYK suppression to augment the anti‐leukaemic effects of FLT3 inhibition in mutant FLT3‐positive leukaemia, we were interested in exploring the combination of FLT3 and SYK suppression in the context of mutant CBL‐positive leukaemia, which is characterized by aberrant FLT3 signalling.

Here, using these cell lines and primary mutant CBL‐positive patient cells, as well as murine xenografts, we performed a side‐by‐side comparison of highly targeted and broad‐spectrum FLT3 inhibitors in late‐stage clinical development or FDA approved for mutant FLT3‐positive AML and demonstrated their ability to suppress mutant CBL‐positive AML proliferation and viability. Furthermore, our studies show that SYK suppression potentiates the anti‐leukaemic activity of FLT3 inhibition in cell line‐based models of mutant CBL‐positive leukaemia.

## 2 MATERIALS AND METHODS

### 2.1 Cell lines

Ba/F3 (interleukin [IL]‐3‐dependent murine pro‐B) cells engineered to express wt FLT3, wt FLT3+wt CBL, wt FLT3+CBL.Ins(SK366), wt FLT3+CBL.Y371H and wt FLT3+CBL.ΔY371 were developed as previously described.^12^ MOLM14 cells were provided to us by Dr Scott Armstrong (Dana‐Farber Cancer Institute, MA). MV4‐11 cells were obtained from Dr Anthony Letai (Dana‐Farber Cancer Institute, MA). Kasumi‐1‐luc+, SKNO‐1‐luc+ and NB4‐luc+ cells were gifts from Dr Andrew Kung (Memorial Sloan Kettering Cancer Center, NY). HEL, HL60 and K052 cell lines were purchased from ATCC. SKM‐1, NOMO‐1 and OCI‐AML3 cell lines were obtained from Dr Gary Gilliland (Fred Hutchinson Cancer Research Center, WA).

Mutant CBL‐expressing Ba/F3 cells and all human cell lines were cultured with 5% CO_2_ at 37°C, at a concentration of 2 × 10^5^ to 5 × 10^5^ in RPMI 1640 (Thermo Fisher Scientific) with 10% foetal bovine serum (FBS) and supplemented with 1% penicillin/streptomycin. Parental Ba/F3 cells, Ba/F3 cells expressing wt FLT3 and Ba/F3 cells expressing wt FLT3+wt CBL were cultured in RPMI with 10% FBS and supplemented with 1% penicillin/streptomycin and 20% WEHI‐conditioned media (as a source of IL‐3).

Cell line authentication is described in the Appendix S1.

### 2.2 AML patient cells

Details are provided in the Appendix S1.

### 2.3 Chemical compounds and biologic reagents

Details are provided in the Appendix S1.

### 2.4 Cell proliferation studies

Details are provided in the Appendix S1.

### 2.5 Immunoblotting and immunoprecipitation

Protein lysate preparation, immunoblotting and immunoprecipitation were carried out as has been previously described.^13^

### 2.6 Antibodies

Antibodies purchased from Cell Signaling Technology were used at a dilution of 1:1000 and include beta‐tubulin (rabbit polyclonal, #2146), phospho‐AKT (Ser 473) (D9E) XP(R) (rabbit mAb, #4060), phospho‐p44/42 MAPK (T202/Y204) (rabbit, #9101), phospho‐S6 ribosomal protein (S235/236) (D57.2.2E) XP (R) (rabbit mAb, #4858), phospho‐Zap‐70 (Tyr319)/SYK (Tyr352) (rabbit, #2701), total AKT (rabbit, #9272), total MAPK (mouse, #9107), total S6 (rabbit, #2217) and total Syk (D3Z1E) XP (rabbit mAb, #13198). Anti‐GAPDH (D16H‐11) XP (R) (rabbit mAb, #5174) (Cell Signaling Technology) was used at a dilution of 1:3000. FLT3/Flk‐2 (C‐20) (sc‐479) was purchased from Santa Cruz Biotechnology, Inc and used at 1:1000 for immunoblotting. Anti‐pTyr (mouse, clone 4G10) was purchased from Upstate MilliporeSigma and was used at 1:1000 in the presence of 4% BSA.

### 2.7 Drug combination studies

Details about drug combination studies, which employed the method of Chou and Talalay,^23^ are provided in the Appendix S1.

### 2.8 Non‐invasive in vivo bioluminescence study

All animal studies were performed according to protocols approved by the Dana‐Farber Cancer Institute's Institutional Animal Care and Use Committee.

Bioluminescence imaging was carried out as previously described.^24^ Briefly, Ba/F3.FLT3(wt).CBL.Ins (SK366)‐luc+ cells suspended in PBS were implanted intravenously (1.5 × 10^6^ cells/mouse) in the female NCr nude mice (7 weeks of age; Taconic, NY). Animals were randomized 3 days post‐implantation using total flux values (sum of prone and supine bioluminescence values) into vehicle control and midostaurin, 100 mg/kg once daily by oral gavage for 21 days (n = 9‐11/group). Bioluminescence imaging was performed once weekly after treatment initiation, and bodyweights were measured twice weekly.

Midostaurin was formulated as a pre‐concentrate/microemulsion with 5% drug powder, 34% Vit E TPGS, 42.5% PEG400, 8.5% corn oil and 10% ethanol. The pre‐concentrate was then dissolved in purified water at a 24:76 ratio on the day of treatment. Stocks of midostaurin were purchased from LC Laboratories and MedChemExpress.

For all in vivo studies, *P* < .05 was considered to be statistically significant. The data had similar variance and met the assumptions of the tests carried out. For in vivo studies investigating the single‐agent effects of midostaurin, the Mann‐Whitney test (two‐tailed) was carried out to assess differences in leukaemia burden between vehicle and drug‐treated mice and the Gehan‐Breslow‐Wilcoxon test was carried out for survival curve comparisons.

### 2.9 RNA sequencing analysis

Total RNA was isolated from midostaurin‐ and DMSO‐treated cells using TRIzol (Ambion by Life Technologies) followed by an RNeasy cleanup step (RNAeasy kit; Qiagen). Library preparation, sequencing of RNA (RNAseq), and changes in gene expression were performed at the Molecular Biology Core Facility (DFCI). Reads were aligned against the mouse genome; *P*‐values were calculated from raw counts, and false discovery rate (FDR) values were calculated using the method of Benjamini and Hochberg.^25^ Gene set enrichment analysis (GSEA)^26^ was performed against the h.all.v6.2.symbols.gmt signature database.

## 3 RESULTS

**Figure 1:**
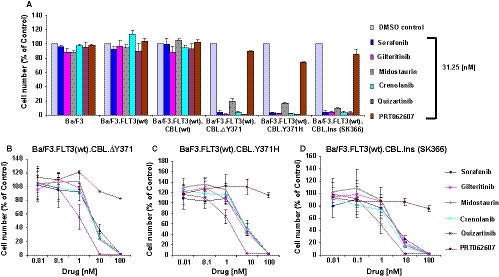
Comparison of the potencies of targeted TKIs against mutant CBL‐expressing cell lines. A, Inhibitors were tested in a proliferation assay in parallel for 3 d (effects of 31.25 nmol/L of each inhibitor shown). Included as controls were growth factor‐dependent parental Ba/F3, as well as growth factor‐dependent Ba/F3 cells expressing wt human FLT3 (Ba/F3.FLT3) and growth factor‐dependent Ba/F3 cells co‐expressing wt human FLT3 and wt human CBL (Ba/F3.FLT3(wt).CBL). All three cell lines not expressing mutant CBL were cultured and tested in the presence of 20% WEHI‐conditioned media, used as a source of IL‐3. Cell lines were treated with sorafenib, gilteritinib, midostaurin, crenolanib, quizartinib and PRT062607. B‐D, Inhibitors were tested in a proliferation assay in parallel for 3 d against Ba/F3.FLT3(wt).CBL.ΔY371 (B), BaF3.FLT3(wt).CBL.Y371H (C) or Ba/F3.FLT3(wt).CBL.Ins (SK366) (D)

Given the demonstrated effectiveness of FLT3 inhibitors against leukaemia characterized by mutated CBL and consequent increased expression of wt FLT3, we were interested in exploring this phenomenon more thoroughly across a panel of widely investigated FLT3 inhibitors in late‐stage clinical development or that have been FDA approved for mutant FLT3‐positive AML. We tested and compared a range of concentrations of sorafenib, gilteritinib, midostaurin, crenolanib and quizartinib against growth factor‐independent Ba/F3 cells engineered to co‐express human wt FLT3 and mutated human CBL (ΔY371, Y371H or Ins(SK366)), as well as growth factor‐dependent parental Ba/F3 cells, Ba/F3 cells engineered to express wt human FLT3 or Ba/F3 cells engineered to co‐express both wt human FLT3 and wt human CBL. As shown in Figure 1A, treatment with 31.25 nmol/L of each FLT3 inhibitor led to a dramatic inhibition of cell proliferation of all three mutant CBL‐expressing cell lines, however, there was little anti‐proliferative effect observed on the growth factor‐dependent cell lines (targeted inhibition of growth of mutant CBL‐expressing cells was observed at concentrations less than 500‐1000 nmol/L) (Figure S1). In comparison with the FLT3 inhibitors, the targeted SYK inhibitors, PRT062607 (P505‐15, B11B057)^27^ and entospletinib (GS‐9973) (Gilead Sciences),^28^ were considerably less effective in inhibiting the proliferation of mutant CBL‐positive cell lines (Figure 1A‐D, Figures S1 and S2).

**Figure 2:**
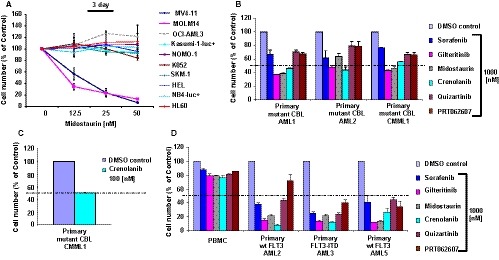
Comparison of the potencies of targeted TKIs against wt FLT3, mutant FLT3 and mutant CBL‐expressing human AML cell lines and primary AML cells. A, Inhibitors were tested in parallel in a proliferation assay for 3 d against human AML cell lines. MOLM14 expresses FLT3‐ITD and mutant CBL. B‐D, Inhibitors were tested in parallel in a proliferation assay for 3 d against primary AML or CMML cells expressing mutant CBL/wt FLT3 (B, C) or human primary AML cells expressing wt FLT3 and mutant FLT3, with normal PBMCs tested as a control (D). Primary mutant CBL AML1 (52% blasts): CBL mutation: D390V. Primary mutant CBL AML2 (3% blasts): CBL mutation: C416R. Primary mutant CBL CMML1 (<5% blasts; CMML is divided into two classifications based on cell counts in the blood and bone marrow. CMML‐1 is characterized by blasts that make up less than 5% of white cells in blood and less than 10% of cells in bone marrow. CMML‐2 is characterized by blasts that make up 5%‐20% of white cells in blood or 10%‐20% of cells in bone marrow): CBL mutations: I383M, C384Y. Primary wt FLT3 AML2 (95% blasts); Primary FLT3‐ITD AML3 (99% blasts); Primary wt FLT3 AML5 (90% blasts). Assay conditions: Primary samples were cultured in RPMI or DMEM supplemented with 10% FBS and 1% pen/strep, with no added cytokines. Cell viability prior to seeding was 98%, as samples were Ficoll‐Paque Plus purified prior to testing in the assay. After 72 h, AML patient cell death is maximum 10%‐12% in the culture system

The murine mutant CBL and wt FLT3‐expressing cell lines provide a relatively simplistic and clean system with which we were able to directly measure effects of FLT3 inhibition, alone and combined with SYK inhibition. We were interested in seeing whether or not these effects were able to be generalized to human AML cell lines and primary AML cells. As expected, midostaurin notably most potently inhibits human AML cell lines, such as MV4‐11 and MOLM14, which express FLT3‐ITD, as compared to other human AML cell lines that express wt FLT3 and that are driven by other oncogenes (Figure 2A). MOLM14 is unique in that it co‐expresses FLT3‐ITD and mutant CBL (monoallelic CBL deletion transcript identified),^2^ however, due to the co‐expression of both oncogenes, growth‐suppressive effects due to inhibition of oncogenic FLT3 cannot be separated from any drug effects that may be associated with mutant CBL expression in these cells.

We next investigated the effects of our panel of kinase inhibitors on primary AML and CMML cells, including those expressing mutant CBL, as well as those expressing wt FLT3/wt CBL, and mutant FLT3/wt CBL. Normal PBMCs acquired from a donor were tested as a control. The wt FLT3/wt CBL and mutant FLT3/wt CBL primary cells generally showed more sensitivity to the inhibitors (at 1000 nmol/L) than the mutant CBL‐expressing AML samples (Figure 2B,D). One exception was a mutant CBL‐positive CMML sample, which showed a loss of around 50% viability with only 100 nmol/L crenolanib (Figure 2C). However, it is important to note that gilteritinib, midostaurin and crenolanib killed over 50% of mutant CBL‐expressing cells, compared with around 70%‐80% of wt FLT3/wt CBL‐expressing AML or mutant FLT3/wt CBL‐expressing AML. In contrast, less than 20% of normal PBMCs were killed by these drugs, suggesting that drug effects were selective for transformed primary patient cells. Table S1 shows a number of genetic mutations that were identified in the mutant CBL‐positive and wt CBL‐expressing samples that may influence the sensitivity or resiliency of these samples to the effects of the kinase inhibitors.

As CBL ubiquitin ligase activity has been implicated in the negative regulation of both SYK and FLT3,^9^, ^10^ and inactivation of CBL via mutation would be expected to impair this regulation, we were interested in measuring the expression levels and activity of the two proteins in mutant CBL‐expressing cells to gain a better understanding of the sensitivity of these cells to FLT3 inhibitors. Specifically, we compared levels of human wt FLT3 and endogenous mouse SYK protein levels, as well as phosphorylation status, between Ba/F3.FLT3(wt) cells and Ba/F3 cells co‐expressing mutant CBL+wt FLT3. A much higher level of wt human FLT3 expression was observed in Ba/F3.FLT3(wt).CBL.Y371H, Ba/F3.FLT3(wt).CBL.Ins (SK366) and Ba/F3.FLT3(wt).CBL.ΔY371 cells as compared to Ba/F3‐FLT3(wt) cells, irrespective of whether Ba/F3.FLT3(wt) cells were cultured overnight in the presence or absence of WEHI‐conditioned media (as a source of IL‐3) (Figure S3A‐C). In contrast, while there was no change in levels of endogenous mouse SYK total protein in mutant CBL‐expressing cells as compared to control cells, SYK phosphorylation was enhanced in mutant CBL‐positive cells (Figure S3D). Increased SYK phosphorylation in mutant CBL‐expressing cells, which are characterized by hyperactivated wt FLT3, may be reflective of the transactivation and cooperativity between highly activated SYK and constitutively activated mutant FLT3 observed in AML patients.^21^ The increases in levels/activity of FLT3 in mutant CBL‐expressing cells may be due to failure of mutated CBL to function properly as an E3 ubiquitin ligase^9^, ^10^ and may also explain the higher susceptibility of these cells to FLT3 inhibitor treatment. Although differences in transfection efficiency cannot be completely ruled out as contributing to these differences, the observed elevated wt FLT3 expression in mutant CBL‐expressing cells is consistent with and supportive of previously published studies focused on effects of mutant CBL in leukaemia.^2^, ^8^, ^9^, ^12^

**Figure 3:**
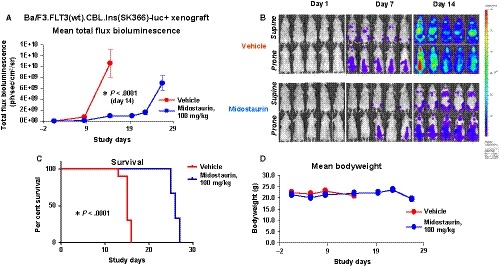
Effects of midostaurin in vivo against mice harbouring Ba/F3.FLT3(wt).CBL.Ins (SK366)‐luc+ cells. A, Measure of leukaemia burden in vehicle‐ vs midostaurin‐treated mice for Ba/F3.FLT3(wt).CBL.Ins (SK366) model. Vehicle‐treated mice were administered vehicle by oral gavage once daily × 28 d. Midostaurin‐treated mice were administered 100 mg/kg midostaurin by oral gavage once daily × 28 d. The Mann‐Whitney test (two‐tailed) was carried out for CBL.Ins (SK366) BLI comparisons. *P* < .0001 for day 14 for the CBL.Ins (SK366) model. B, Effects of midostaurin in vivo against mice harbouring Ba/F3.FLT3(wt).CBL.Ins (SK366)‐luc+ cells. Supine and Prone (High Scale), Days 1‐14. Representative Images (n = 5). C, Measure of survival of vehicle‐ vs midostaurin‐treated mice for CBL.Ins (SK366) model. Gehan‐Breslow‐Wilcoxon test was carried out for CBL.Ins (SK366) survival curve comparisons. *P* < .0001. D, Mean weights of mice from the CBL.Ins (SK366) murine model

**Figure 4:**
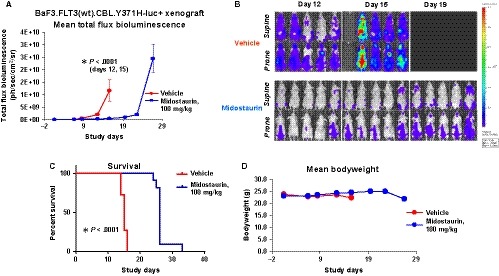
Effects of midostaurin in vivo against mice harbouring Ba/F3.FLT3(wt).CBL.Y371H‐luc+ cells. A, Measure of leukaemia burden in vehicle‐ vs midostaurin‐treated mice for CBL.Y371H model. Vehicle‐treated mice were administered vehicle by oral gavage once daily × 28 d. Midostaurin‐treated mice were administered 100 mg/kg midostaurin by oral gavage once daily × 28 d. The Mann‐Whitney test (two‐tailed) was carried out for CBL.Y371H BLI comparisons. *P* < .0001 for days 12 and 15 for the CBL.Y371H model. B, Effects of midostaurin in vivo against mice harbouring Ba/F3.FLT3(wt).CBL.Y371H‐luc+ cells. Supine and Prone (High Scale), Days 12‐19. Representative Images (n = 5). C, Measure of survival of vehicle‐ vs midostaurin‐treated mice for CBL.Y371H model. Gehan‐Breslow‐Wilcoxon test was carried out for CBL.Y371H survival curve comparisons. *P* < .0001. D, Mean weights of mice from the CBL.Y371H murine model

We next investigated the ability of midostaurin to inhibit the proliferation of luciferase‐expressing Ba/F3.FLT3(wt).CBL.Ins (SK366) cells and Ba/F3.FLT3(wt).CBL.Y371H cells in vivo*.* Treatment of NCr nude mice harbouring each cell line with 100 mg/kg midostaurin administered orally once daily for 21 days led to a significantly delayed leukaemic cell growth (Ba/F3.FLT3(wt).CBL.Ins (SK366) model; *P* < .0001 day 14 and Ba/F3.FLT3(wt).CBL.Y371H model; *P* < .0001 days 12 and 15) (Figures 3A,B and 4A,B, Figures S4 and S5) and significantly prolonged survival (Ba/F3.FLT3(wt).CBL.Ins (SK366) model; *P* < .0001 and Ba/F3.FLT3(wt).CBL.Y371H model; *P* < .0001) (Figures 3C and 4C) as compared to vehicle‐treated control mice. Midostaurin treatment was well tolerated (Figures 3D and 4D).

**Figure 5:**
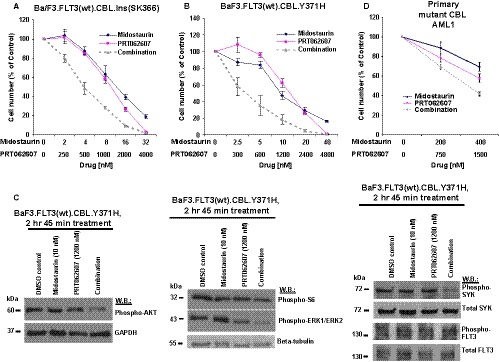
Effects of midostaurin, alone and combined with PRT062607, on proliferation and on the activity of signalling molecules downstream of FLT3 in mutant CBL‐positive leukaemia cells. A‐B, Midostaurin and PRT062607 were tested in a proliferation assay alone and in combination for 3 d against mutant CBL‐expressing Ba/F3 cell lines. C, Midostaurin was tested alone and in combination with PRT062607 for effects on the activity of signalling molecules downstream of FLT3 (left and middle panels) or on SYK or FLT3 directly (right‐hand panel) in Ba/F3 cells co‐expressing human wt FLT3 and mutant CBL (Y371H). Cells were treated for 2 h and 45 min prior to generation of protein lysate. D, Midostaurin and PRT062607 were tested in a proliferation assay alone and in combination for 3 d against mutant CBL‐expressing primary AML1

**Figure 6:**
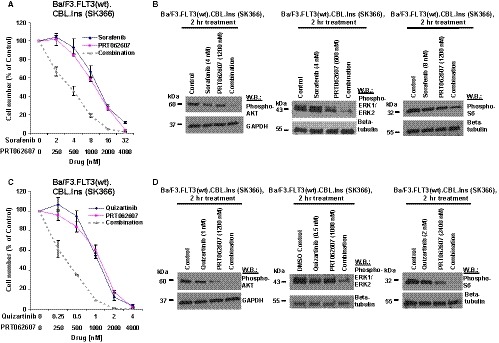
Effects of TKIs alone and in combination with PRT062607 against Ba/F3.FLT3(wt).CBL.Ins (SK366) cells. A, C TKIs were tested in a proliferation assay alone and in combination with PRT062607 for 3 d against Ba/F3.FLT3(wt).CBL.Ins (SK366) cells. B, D, Sorafenib or quizartinib was tested alone and in combination with PRT062607 for effects on the activity of signalling molecules downstream of FLT3 in Ba/F3 cells co‐expressing human wt FLT3 and mutant CBL (insert, Ins [SK366]). Cells were treated for 2 h

Considering the previously demonstrated ability of SYK inhibitors to potentiate the inhibitory activity of FLT3 inhibitors against oncogenic FLT3‐positive leukaemia, we hypothesized that the combination of FLT3 and SYK inhibition might be similarly more effective against cells engineered to co‐express mutant CBL and wt FLT3, as compared to FLT3 inhibition alone. The ability of the SYK inhibitor, PRT062607, to potentiate the effects of midostaurin against Ba/F3.FLT3(wt).CBL.Y371H, Ba/F3.FLT3(wt).CBL.Ins(SK366), or Ba/F3.FLT3(wt).CBL.ΔY371 cells, as evidenced by a leftward shift in the combination curves, is shown in Figure 5A,B and Figure S6. Similarly, PRT062607 augmented the anti‐proliferative activity of sorafenib or quizartinib against mutant CBL‐ and wt FLT3‐expressing cells (Figure 6A,C and Figure S7A,C). Combination indices, generated by Calcusyn software, are mostly lower than 1.1, which is the cut‐off for nearly additive effects, and are indicative of different levels of synergy for the combination of all tested FLT3 inhibitors and SYK suppression by PRT062607 or entospletinib (Table 1, upper panel). It is possible that the higher levels and or activity of both FLT3 and SYK in mutant CBL‐expressing cells might explain why SYK inhibition potentiates the effects of FLT3 inhibitors against these cells.

**Table 1:**
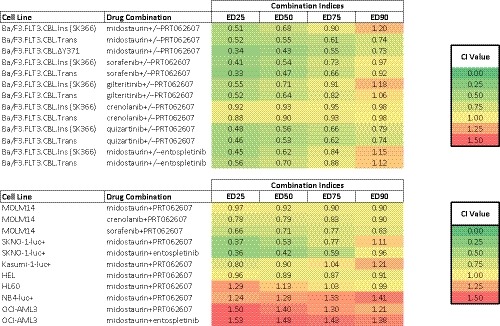
Combination indices generated by Calcusyn software for FLT3 inhibitors combined with PRT062607 or entospletinib against mutant CBL‐expressing Ba/F3 cells (top panel) or against human AML cell lines (bottom panel)

Note

Proliferation assays/combination studies were carried out for 3 d. Calcusyn combination indices can be interpreted as follows: CI values <0.10 indicate very strong synergism; values 0.10‐0.30 indicate strong synergism; values 0.30‐0.70 indicate synergism; values 0.70‐0.85 indicate moderate synergism; values 0.85‐0.90 indicate slight synergism; values 0.90‐1.10 indicate nearly additive effects; values 1.10‐1.20 indicate slight antagonism; values 1.20‐1.45 indicate moderate antagonism; values 1.45‐3.30 indicate antagonism; values 3.30‐10.0 indicate strong antagonism; values >10.0 indicate very strong antagonism.

Investigation of drug effects on signalling molecules downstream of FLT3 in Ba/F3.FLT3(wt).CBL.Ins (SK366) and Ba/F3.FLT3(wt).CBL.Y371H cell lines generally showed increased suppression of phosphorylation of AKT, ERK1/ERK2 and S6 by the combination of FLT3 inhibitors and SYK inhibition as compared to single agents (Figures 5C and 6B,D, Figure [Link], [Link]B,D,E). These results suggest the likely involvement of the PI3K/AKT and MAPK signalling pathways in the observed synergistic interaction between FLT3 and SYK inhibition in this system.

In addition to looking at downstream effectors, we also investigated effects of midostaurin or quizartinib combined with PRT062607 against SYK and FLT3 directly. A combination effect was observed between midostaurin and PRT062607 against phospho‐SYK, presumably due to the fact that SYK is a target of both midostaurin and PRT062607 (Figure 5C). In contrast, there was no combination effect observed between quizartinib and PRT062607, which may be due to the fact that SYK, while a target of PRT062607, is not a target of quizartinib (Figure S7F). Neither midostaurin+PRT062607 nor quizartinib+PRT062607 showed combination effects against phospho‐FLT3, which may be due to the fact that although FLT3 is a target of midostaurin and quizartinib, FLT3 is not a target of PRT062607 (Fig 5C and Fig S7F). Drug concentrations used for these combination studies were derived from dose‐response studies carried out with each inhibitor (Figures [Link], [Link], [Link] and [Link], [Link], [Link]).

The effects of midostaurin and FLT3 inhibitors, alone and combined, were tested against mutant FLT3/mutant CBL‐expressing MOLM14. We observed potentiation of the effects of both midostaurin, crenolanib and sorafenib by PRT062607, with combination indices suggestive of additive to synergistic effects across a range of concentrations (Figure S10A,B and Table 1, lower panel). Anti‐proliferative effects of midostaurin+PRT062607 correlated with decreases in phospho‐AKT and phospho‐S6 (Figure S10C). For comparison, we also tested the effects of midostaurin combined with PRT062607 or entospletinib against a panel of wt CBL‐expressing human AML cell lines. We observed additive to synergistic effects for the combinations against cell lines, including SKNO‐1‐luc+, Kasumi‐1‐luc+, HEL and HL60, whereas combinations were antagonistic against NB4‐luc+ and OCI‐AML3 cells (Figure S10D,E and Table 1, lower panel). In addition to cell lines, we tested midostaurin and PRT062607, alone and combined, against mutant CBL‐positive primary AML cells (AML1). The effects of midostaurin were potentiated by PRT062607, with more killing of the primary patient cells observed with the drug combination than with either agent alone (Figure 5D).

Taken together, our results support the idea that mutant CBL‐expressing AML, characterized by hyperactivation of FLT3 signalling, is susceptible to killing by similar approaches used for mutant FLT3‐positive AML. This includes treatment with targeted FLT3 inhibitors representative of a wide range of selectivity for FLT3 as a target, as well as combination therapy based on both FLT3 and SYK inhibition.

## 4 DISCUSSION

Patients with myeloid neoplasms having mutations in CBL generally have a poor prognosis. For MDS patients, mutations in CBL, IDH2, DNMT3A, TP53 or ASXL1, individually, are associated with shorter survival, with a 3‐year survival rate of only 27% for MDS/MPN patients characterized by having activating mutations of CBL.^29^, ^30^ Thus, novel treatment strategies that can be readily implemented in the clinic are needed.

The direct targeting of CBL is challenging, as with inactivating mutations finding direct binders is unlikely to be beneficial. Furthermore, there are few successful examples of ligands for ligases. As such, investigating the effectiveness of targeting upstream/downstream factors related to mutant CBL is important to develop a therapeutic strategy.

There are striking similarities between the effects of mutant CBL and those of mutated FLT3 on intracellular signalling pathways, namely hyperactivation of signalling molecules downstream of FLT3 that lead to cellular transformation. Therefore, it would be expected that targeted inhibitors of FLT3 would be an effective therapy for CBL mutant‐driven leukaemia as well as for mutant FLT3‐positive leukaemia.

It has been proposed that, due to observations such as constitutive phosphorylation of STAT‐5 in FLT3‐ITD cells but not in FLT3 wt cells, the signalling mechanisms of mutant FLT3 differ at least to a degree from the signalling mechanisms of wt FLT3.^31^ However, in the case of mutant CBL‐expressing cells, wt FLT3 is overexpressed and hyperstimulated and thus from a signalling standpoint resembles FLT3‐ITD more than it does wt FLT3. We present the following hypothesis to at least partly explain this phenomenon: As mutant CBL renders CBL functionally inactive, there is a decrease in ubiquitination of FLT3 and a consequent increase in FLT3 levels and activity. Hyperactivated FLT3, similar to constitutively activated FLT3‐ITD, thus leads to the activation of downstream signalling molecules, such as AKT, ERK pathways and STAT5. We acknowledge, however, that the process may be more complex than what we are proposing, and CBL itself may phosphorylate FLT3 through a direct interaction,^12^ or the CBL mutant may activate signalling pathways through other substrates independently of FLT3, such as JAK2,^32^, ^33^ a possibility that warrants testing of targeted JAK2 inhibitors in cell growth assays and AKT/ERK/STAT signalling studies.

Here, via a thorough and extensive comparison of the anti‐leukaemic potential of a panel of FLT3 inhibitors that have either been FDA approved (midostaurin and gilteritinib) or that are in late‐stage clinical development (crenolanib, quizartinib and sorafenib), we show that all FLT3 inhibitors, regardless of their broad or narrow range of targeted activity, are potent inhibitors of growth of mutant CBL‐positive leukaemia, with some, like crenolanib, gilteritinib and midostaurin, showing more activity than others against primary mutant CBL‐positive leukaemia. FLT3 inhibitors at a concentration of 1000 nmol/L were effective in inhibiting growth of primary AML and CMML cells harbouring not only mutant CBL, but also other oncogenes, including ASXL1, RUNX1, IDH2, SRSF2, STAG2, AXL1 and TET2 (Table S1). Crenolanib displayed the highest potency of all the FLT3 inhibitors against primary CMML expressing two different CBL mutations (CBL (1383M) and CBL (C384Y)), with an IC50 of around 100 nmol/L.

In mice, a mutation in the RING finger domain of CBL causes development of a myeloproliferative disease (MPD) that progresses to leukaemia.^34^ MPD driven by wt FLT3 results from a CBL RING finger mutation and is suppressed by treatment with quizartinib.^35^ Similarly, transplantation of murine bone marrow cells infected with a retrovirus expressing a FLT3‐ITD mutant results in the development of a rapidly lethal MPD in mice,^36^ and we and others have shown that mice harbouring oncogenic FLT3 benefit from treatment with FLT3 inhibitors such as midostaurin or gilteritinib.^13^, ^37^ Thus, the parallels between mutant CBL‐positive and oncogenic FLT3‐positive disease are clear, primarily in terms of the shared ability of each to induce MPD and their susceptibility to FLT3 inhibition. Here, we show the ability of midostaurin to rapidly and potently inhibit the in vivo growth of mutant CBL‐positive leukaemia cells in two distinct models, highlighting the potential for FLT3 inhibitors like midostaurin that are approved and are in current clinical use for patients with aberrant FLT3 signalling.

Clinical studies with midostaurin have shown that its effects as a single agent are at best partial and transient, and for patients to achieve maximum clinical benefit midostaurin must be administered in combination with other anti‐cancer drugs.^14^, ^38^ Highly activated SYK has been observed to be enriched in FLT3‐ITD‐positive patient AML, and its cooperation with oncogenic FLT3 leads to activation of MYC transcriptional programmes.^21^ Consistent with this, in the present study we observed higher SYK activity in mutant CBL‐expressing cells relative to control cells. Mutant CBL‐expressing cells were also noted to more robustly express FLT3 and showed increased phosphorylation of FLT3, relative to control cells. As we have previously shown that the inhibitory effect of midostaurin can be further enhanced by additional SYK inhibition against mutant FLT3‐positive AML,^22^ we were particularly interested in investigating the synergizing potential of FLT3 and SYK inhibition in the context of mutant CBL‐positive leukaemia, given our observations.

We have previously shown that midostaurin, a multitargeted kinase inhibitor, is an inhibitor of both SYK and FLT3; we accomplished this using Ba/F3 cells expressing mutant SYK fusion genes and FLT3‐ITD.^22^ In addition, we have found midostaurin to be unique among the FLT3 inhibitors tested here in terms of its ability to inhibit SYK as well as FLT3 (unpublished results). Here, we expand on our previous findings to show that, because of the similarities between FLT3 signalling in mutant FLT3‐ and mutant CBL‐expressing AML, the inhibitory effects of FLT3 inhibitors can similarly be potentiated by SYK inhibition in both AML subtypes. Cooperativity and transactivation of FLT3‐ITD and SYK are transforming, and this causes the two proteins, acting jointly, to be viable targets for combination therapy.^21^ In similar fashion, it is possible that hyperactivated wt FLT3 mimics FLT3‐ITD, and it is also plausible that overstimulated FLT3 acts in concert with activated SYK, and this may account for observed synergy between FLT3 inhibition and SYK inhibition. Specifically, we found that additional SYK inhibition, both that of PRT062607 and the clinically investigated SYK inhibitor, entospletinib, augments the anti‐leukaemic activity of midostaurin and other FLT3 inhibitors regardless of their respective SYK‐targeting activity in our cell line models of mutant CBL‐positive leukaemia as well as mutant FLT3/mutant CBL‐positive MOLM14 cells. Signalling molecules activated and downstream of FLT3, including phospho‐AKT, phospho‐ERK1/ERK2 and phospho‐S6, were suppressed to a greater extent by the combination of SYK inhibition and FLT3 inhibition as compared to single agents, suggesting that blockade of associated pathways contributes to the observed synergy.

In an attempt to better understand the mechanisms underlying the effects of midostaurin on mutant FLT3‐ vs mutant CBL‐expressing cells, we performed RNAseq analysis. We compared signalling pathway gene regulation between Ba/F3.FLT3(wt), CBL. FLT3(wt).Y371H and Ba/F3‐FLT3‐ITD cells treated under identical culture conditions with DMSO or 50 nmol/L midostaurin for 24 hours. As expected, few changes (>2‐fold change in gene expression, *P* < .05) were observed in unstimulated Ba/F3 expressing wt FLT3. The expression of only six genes was found to be significant in response to midostaurin treatment (Table S2). In contrast, the expression of 290 genes was altered in midostaurin‐treated Ba/F3‐FLT3‐ITD cells and 648 genes were altered in midostaurin‐treated Ba/F3.FLT3(wt).CBL.Y371H cells. The lack of meaningful change in signalling pathway regulation by midostaurin in Ba/F3.FLT3(wt) cells led us to focus on comparison between mutant FLT3‐ vs mutant CBL‐expressing cells. We identified 15 significantly changed pathways in FLT3‐ITD expression cells and 18 changed pathways in cells expressing both FLT3(wt) and CBL.Y371H. Consistent with a central role of FLT3, we found significant overlap (Figure S11 and Table S2). In particular, pathways previously associated with FLT3 signalling, including MTORC signalling, STAT5 signalling and MYC targets, were found to be similar in both cell systems. These results suggest that factors related to FLT3‐mediated signalling are targeted by midostaurin not only in FLT3‐ITD expressing cells, but also in cells containing mutant CBL, signalling through FLT3wt.

Taken together, our findings suggest that FLT3 inhibitors in advanced stages of development or FDA approved as therapy for mutant FLT3‐positive AML, and representing a wide range of specificity for FLT3, could similarly be considered as treatments for mutant CBL‐positive leukaemia. In addition, synergy observed between targeted FLT3 inhibitors, irrespective of their SYK inhibitory potential, and targeted SYK inhibitors indicate that this combination drug strategy may be efficacious for patients harbouring CBL mutations. These results yield novel insights for possible clinical investigation of targeted therapy for mutant CBL‐positive AML and suggest that therapies beneficial for mutant FLT3‐positive leukaemia may be similarly beneficial for mutant CBL‐positive leukaemia due to the similarities between the two leukaemia subtypes.

## CONFLICT OF INTEREST

James D. Griffin receives funding and has received a royalty payment from Novartis Pharmaceuticals and receives funding from Eli Lilly and Company. Nathanael Gray is a founder, science advisory board member (SAB) and equity holder in Gatekeeper, Syros, Petra, C4, B2S and Soltego. The Gray laboratory receives or has received research funding from Novartis, Takeda, Astellas, Taiho, Janssen, Kinogen, Voronoi, Her2llc, Deerfield and Sanofi. Ellen Weisberg has received a royalty payment from Novartis Pharmaceuticals. Richard Stone does Ad Hoc consulting for and receives clinical research support to Dana‐Farber Cancer Institute from the following companies: Abbvie, Agios, Arog and Novartis. He does Ad Hoc consulting for the following companies: Astrazeneca, Cornerstone, Jazz, Daiichi Sankyo, Otsuka/Astex, Pfizer and Stemline. He is on the Advisory Board of the following companies: Actinium, Amgen, Astellas and Macrogenics. He is on the Data Safety and Monitoring Board for the following companies: Argenx, Celgene and Takeda. He is an Ad Hoc Consultant and on the Steering Committee and Data Safety and Monitoring Board for Celgene.

## AUTHOR CONTRIBUTIONS

All authors listed have: (a) made substantial contributions to research design, or the acquisition, analysis or interpretation of data and (b) drafted the paper or revised it critically. All authors have approved the submitted and final version of this manuscript.

## Data Availability

The data that support the findings of this study are available from the corresponding author upon reasonable request.

## References

[1] Saultz JN , Garzon R . Acute myeloid leukemia: a concise review. J Clin Med. 2016;5:E33. 26959069 10.3390/jcm5030033 PMC4810104

[2] Reindl C , Quentmeier H , Petropoulos K , et al. CBL exon 8/9 mutants activate the FLT3 pathway and cluster in core binding factor/11q deletion acute myeloid leukemia/myelodysplastic syndrome subtypes. Clin Cancer Res. 2009;15:2238‐2247. 19276253 10.1158/1078-0432.CCR-08-1325

[3] Haferlach C , Dicker F , Kohlmann A , et al. AML with CBFB‐MYH11 rearrangement demonstrate RAS pathway alterations in 92% of all cases including a high frequency of NF1 deletions. Leukemia. 2010;24:1065‐1069. 20164853 10.1038/leu.2010.22

[4] Masuda S . Point mutations in myelodysplastic syndromes. N Engl J Med. 2011;365:1154. 10.1056/NEJMc1108665 21992130

[5] Murati A , Brecqueville M , Devillier R , Mozziconacci M‐J , Gelsi‐Boyer V , Birnbaum D . Myeloid malignancies: mutations, models and management. BMC Cancer. 2012;12:304. 22823977 10.1186/1471-2407-12-304 PMC3418560

[6] Klampfl T , Milosevic JD , Puda A , et al. Complex patterns of chromosome 11 aberrations in myeloid malignancies target CBL, MLL, DDB1 and LMO2. PLoS ONE. 2013;8:e77819. 24147083 10.1371/journal.pone.0077819 PMC3797696

[7] Dunbar AJ , Gondek LP , O'Keefe CL , et al. 250K single nucleotide polymorphism array karyotyping identifies acquired uniparental disomy and homozygous mutations, including novel missense substitutions of c‐Cbl, in myeloid malignancies. Cancer Res. 2008;68:10349‐10357. 19074904 10.1158/0008-5472.CAN-08-2754 PMC2668538

[8] Sanada M , Suzuki T , Shih L‐Y , et al. Gain‐of‐function of mutated C‐CBL tumour suppressor in myeloid neoplasms. Nature. 2009;460:904‐908. 19620960 10.1038/nature08240

[9] Sargin B , Choudhary C , Crosetto N , et al. Flt3‐dependent transformation by inactivating c‐Cbl mutations in AML. Blood. 2007;110:1004‐1012. 17446348 10.1182/blood-2007-01-066076

[10] Rao N , Ghosh AK , Ota S , et al. The non‐receptor tyrosine kinase Syk is a target of Cbl‐mediated ubiquitylation upon B‐cell receptor stimulation. EMBO J. 2001;20:7085‐7095. 11742985 10.1093/emboj/20.24.7085 PMC125791

[11] Naramura M , Nadeau S , Mohapatra B , et al. Mutant Cbl proteins as oncogenic drivers in myeloproliferative disorders. Oncotarget. 2011;2:245‐250. 21422499 10.18632/oncotarget.233 PMC3134300

[12] Fernandes MS , Reddy MM , Croteau NJ , et al. Novel oncogenic mutations of CBL in human acute myeloid leukemia that activate growth and survival pathways depend on increased metabolism. J Biol Chem. 2010;285:32596‐32605. 20622007 10.1074/jbc.M110.106161 PMC2952262

[13] Weisberg E , Boulton C , Kelly LM , et al. Inhibition of mutant FLT3 receptors in leukemia cells by the small molecule tyrosine kinase inhibitor PKC412. Cancer Cell. 2002;1:433‐443. 12124173 10.1016/s1535-6108(02)00069-7

[14] Stone RM , Mandrekar SJ , Sanford BL , et al. Midostaurin plus chemotherapy for acute myeloid leukemia with a FLT3 mutation. N Engl J Med. 2017;377:454‐464. 28644114 10.1056/NEJMoa1614359 PMC5754190

[15] Thom C . Preliminary data on ASP2215: tolerability and efficacy in acute myeloid leukemia patients. Future Oncol. 2015;11:2499‐2501. 26279055 10.2217/fon.15.188

[16] Lee LY , Hernandez D , Rajkhowa T , et al. Preclinical studies of gilteritinib, a next‐generation FLT3 inhibitor. Blood. 2017;129:257‐260. 27908881 10.1182/blood-2016-10-745133 PMC5234222

[17] Chao Q , Sprankle KG , Grotzfeld RM , et al. Identification of N‐(5‐tert‐butyl‐isoxazol‐3‐yl)‐N’‐{4‐[7‐(2‐morpholin‐4‐yl‐ethoxy)imidazo[2,1‐b][1,3]benzothiazol‐2‐yl]phenyl}urea dihydrochloride (AC220), a uniquely potent, selective, and efficacious FMS‐like tyrosine kinase‐3 (FLT3) inhibitor. J Med Chem. 2009;52:7808‐7816. 19754199 10.1021/jm9007533

[18] Galanis A , Levis M . Inhibition of c‐Kit by tyrosine kinase inhibitors. Haematologica. 2015;100:e77‐e79. 25425690 10.3324/haematol.2014.117028 PMC4349281

[19] Smith CC , Lasater EA , Lin KC , et al. Crenolanib is a selective type I pan‐FLT3 inhibitor. Proc Natl Acad Sci USA. 2014;111:5319‐5324. 24623852 10.1073/pnas.1320661111 PMC3986131

[20] Zhang W , Konopleva M , Shi Y‐X , et al. Mutant FLT3: a direct target of sorafenib in acute myelogenous leukemia. J Natl Cancer Inst. 2008;100:184‐198. 18230792 10.1093/jnci/djm328

[21] Puissant A , Fenouille N , Alexe G , et al. SYK is a critical regulator of FLT3 in acute myeloid leukemia. Cancer Cell. 2014;25:226‐242. 24525236 10.1016/j.ccr.2014.01.022 PMC4106711

[22] Weisberg EL , Puissant A , Stone R , et al. Characterization of midostaurin as a dual inhibitor of FLT3 and SYK and potentiation of FLT3 inhibition against FLT3‐ITD‐driven leukemia harbouring activated SYK kinase. Oncotarget. 2017;8:52026‐52044. 28881711 10.18632/oncotarget.19036 PMC5581010

[23] Chou TC , Talalay P . Quantitative analysis of dose‐effect relationships: the combined effects of multiple drugs or enzyme inhibitors. Adv Enzyme Regul. 1984;22:27‐55. 6382953 10.1016/0065-2571(84)90007-4

[24] Weisberg E , Manley PW , Breitenstein W , et al. Characterization of AMN107, a selective inhibitor of native and mutant Bcr‐Abl. Cancer Cell. 2005;7:129‐141. 15710326 10.1016/j.ccr.2005.01.007

[25] Benjamini Y , Hochberg Y . Controlling the false discovery rate: a practical and powerful approach to multiple testing. J R Stat Soc Series B (Methodol). 1995;57:289‐300.

[26] Mootha VK , Lindgren CM , Eriksson KF , et al. PGC‐1 alpha‐responsive genes involved in oxidative phosphorylation are coordinately downregulated in human diabetes. Nat Genet. 2003;34:267‐273. 12808457 10.1038/ng1180

[27] Hoellenriegel J , Coffey GP , Sinha U , et al. Selective, novel spleen tyrosine kinase (Syk) inhibitors suppress chronic lymphocytic leukemia B‐cell activation and migration. Leukemia. 2012;26:1576‐1583. 22362000 10.1038/leu.2012.24 PMC5459370

[28] Currie KS , Kropf JE , Lee T , et al. Discovery of GS‐9973, a selective and orally efficacious inhibitor of spleen tyrosine kinase. J Med Chem. 2014;57:3856‐3873. 24779514 10.1021/jm500228a

[29] Schwaab J , Ernst T , Erben P , et al. Activating CBL mutations are associated with a distinct MDS/MPN phenotype. Ann Hematol. 2012;91:1713‐1720. 23010802 10.1007/s00277-012-1521-3

[30] Hou H‐A , Tsai C‐H , Lin C‐C , et al. Incorporation of mutations in five genes in the revised International Prognostic Scoring System can improve risk stratification in the patients with myelodysplastic syndrome. Blood Cancer J. 2018;8:39. 29618722 10.1038/s41408-018-0074-7 PMC5884776

[31] Quentmeier H , Reinhardt J , Zaborski M , Drexler HG . FLT3 mutations in acute myeloid leukemia cell lines. Leukemia. 2003;17:120‐124. 12529668 10.1038/sj.leu.2402740

[32] Lv K , Jiang J , Donaghy R , et al. CBL family E3 ubiquitin ligases control JAK2 ubiquitination and stability in hematopoietic stem cells and myeloid malignancies. Genes Dev. 2017;31:1007‐1023. 28611190 10.1101/gad.297135.117 PMC5495118

[33] Tasian SK , Casas JA , Posocco D , et al. Mutation‐specific signaling profiles and kinase inhibitor sensitivities of juvenile myelomonocytic leukemia revealed by induced pluripotent stem cells. Leukemia. 2019;33:181‐190. 29884903 10.1038/s41375-018-0169-y PMC6286697

[34] Rathinam C , Thien CBF , Flavell RA , Langdon WY . Myeloid leukemia development in c‐Cbl RING finger mutant mice is dependent on FLT3 signaling. Cancer Cell. 2010;18:341‐352. 20951944 10.1016/j.ccr.2010.09.008

[35] Taylor SJ , Dagger SA , Thien CBF , Wikstrom ME , Langdon WY . Flt3 inhibitor AC220 is a potent therapy in a mouse model of myeloproliferative disease driven by enhanced wild‐type Flt3 signaling. Blood. 2012;120:4049‐4057. 22990016 10.1182/blood-2012-06-436675

[36] Kelly LM , Liu Q , Kutok JL , Williams IR , Boulton CL , Gilliland DG . FLT3 internal tandem duplication mutations associated with human acute myeloid leukemias induce myeloproliferative disease in a murine bone marrow transplant model. Blood. 2002;99:310‐318. 11756186 10.1182/blood.v99.1.310

[37] Mori M , Kaneko N , Ueno Y , et al. Gilteritinib, a FLT3/AXL inhibitor, shows antileukemic activity in mouse models of FLT3 mutated acute myeloid leukemia. Invest New Drugs. 2017;35:556‐565. 28516360 10.1007/s10637-017-0470-z PMC5613053

[38] Fischer T , Stone RM , DeAngelo DJ , et al. Phase IIB trial of oral Midostaurin (PKC412), the FMS‐like tyrosine kinase 3 receptor (FLT3) and multitargeted kinase inhibitor, in patients with acute myeloid leukemia and high‐risk myelodysplastic syndrome with either wild‐type or mutated FLT3. J Clin Oncol. 2010;28:4339‐4345. 20733134 10.1200/JCO.2010.28.9678 PMC4135183

