# Supplementary figures and images for: The combination of FLT3 and SYK kinase inhibitors is toxic to leukaemia cells with CBL mutations
Source: J Cell Mol Med. 2020 Jan 14;24(3):2145–56. doi: 10.1111/jcmm.14820 (PMC7011134; doi:10.1111/jcmm.14820)

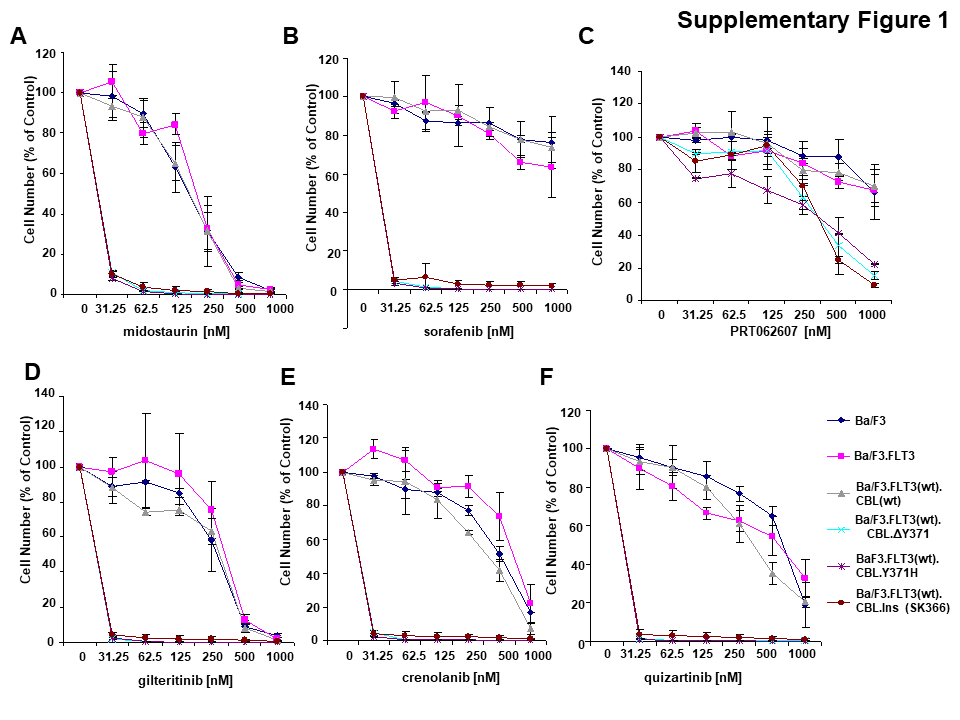

Supplement: Supplementary file 1 [file JCMM-24-2145-s001.TIF]

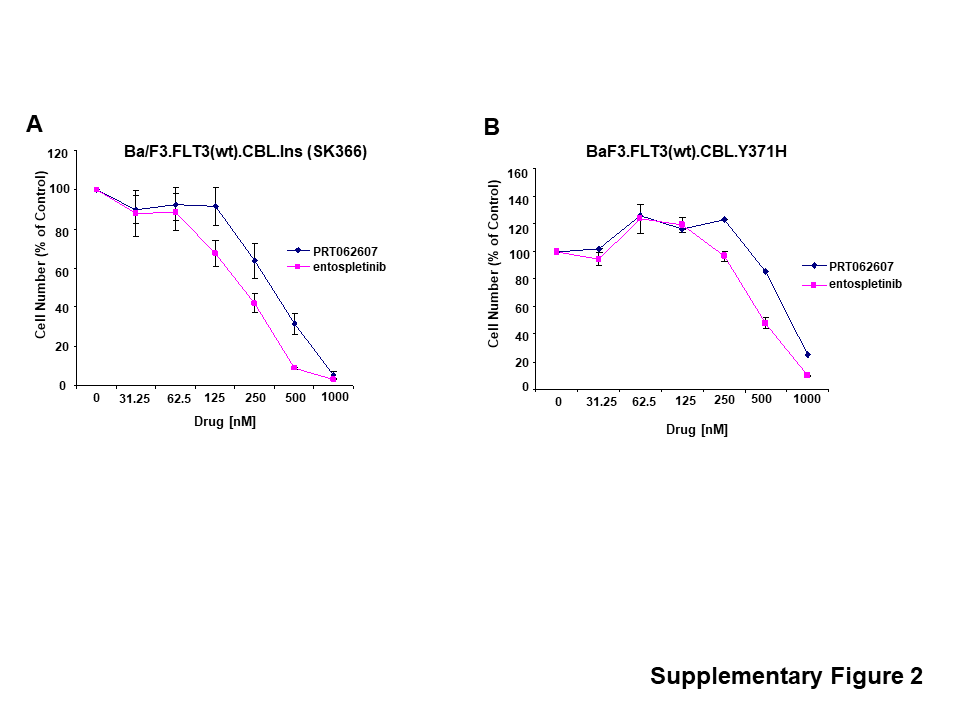

Supplement: Supplementary file 2 [file JCMM-24-2145-s002.TIF]

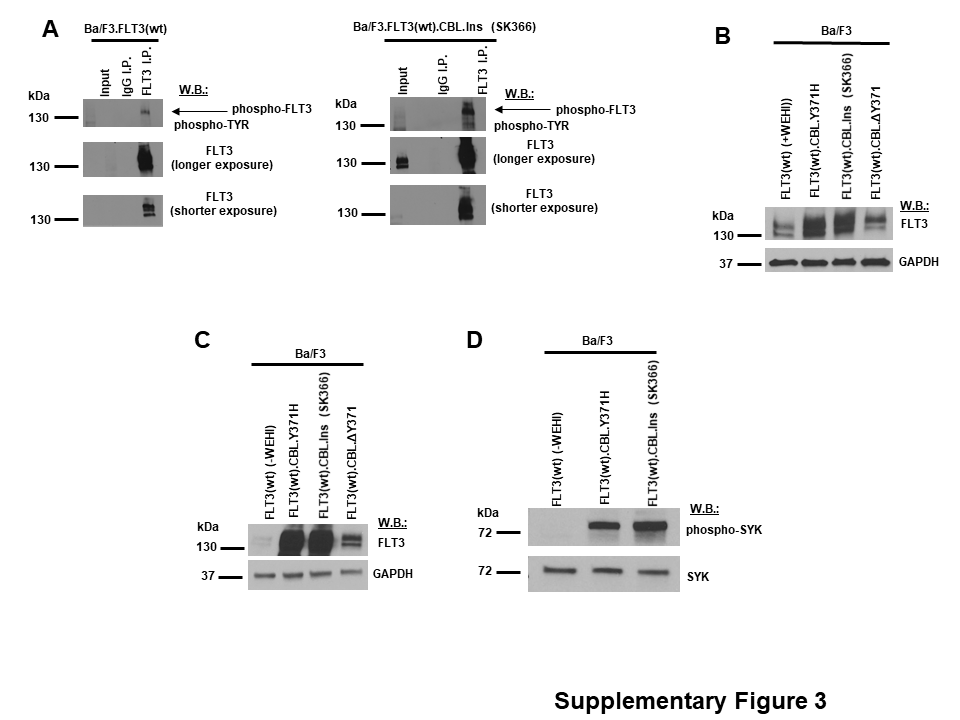

Supplement: Supplementary file 3 [file JCMM-24-2145-s003.TIF]

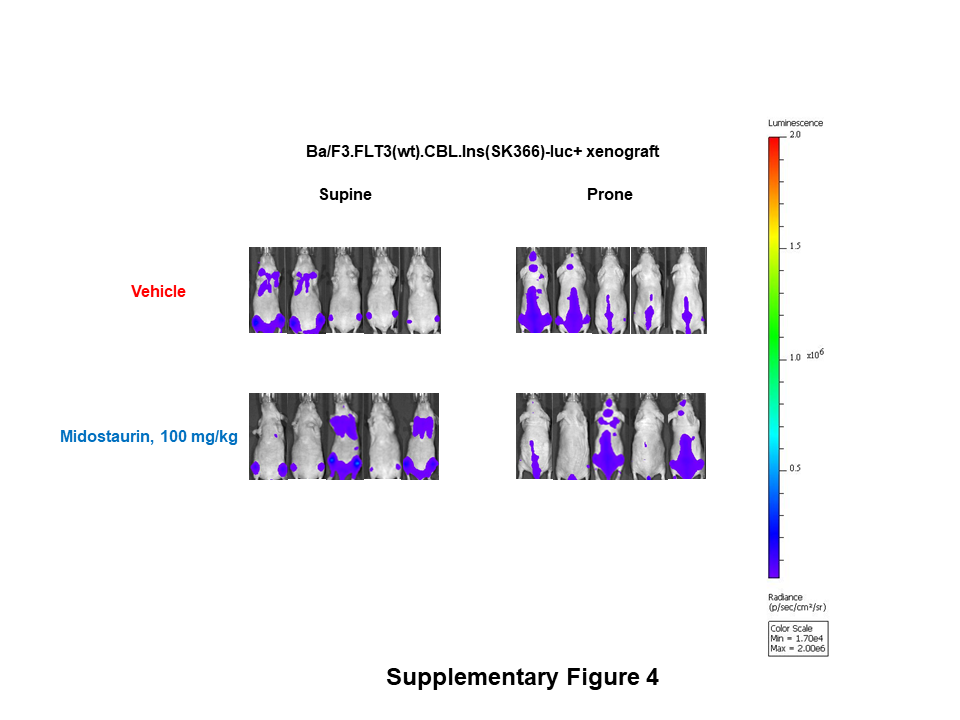

Supplement: Supplementary file 4 [file JCMM-24-2145-s004.TIF]

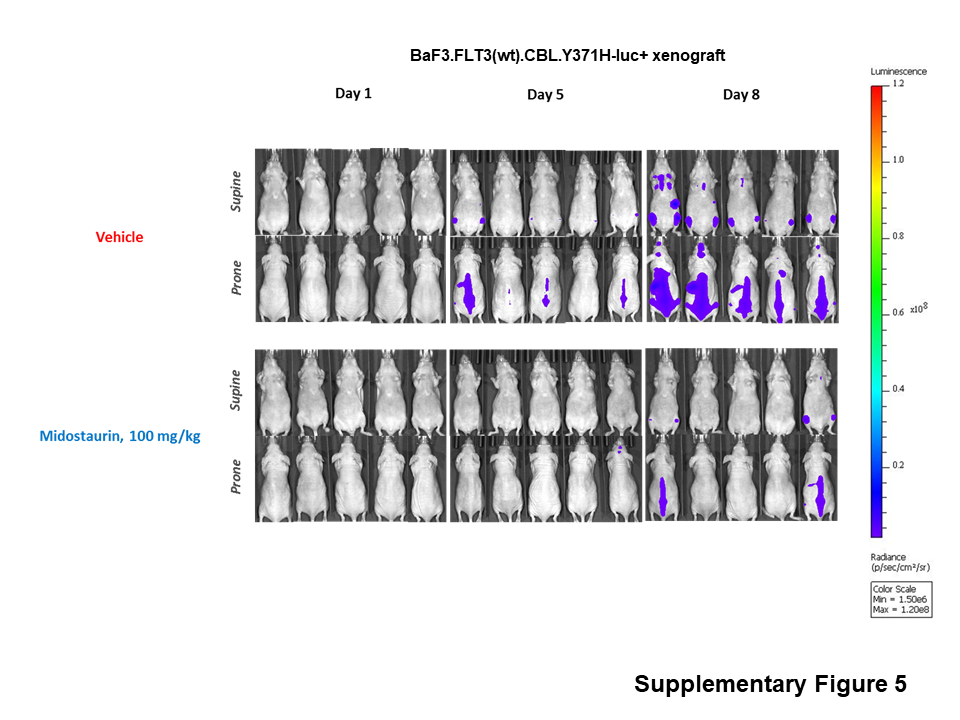

Supplement: Supplementary file 5 [file JCMM-24-2145-s005.TIF]

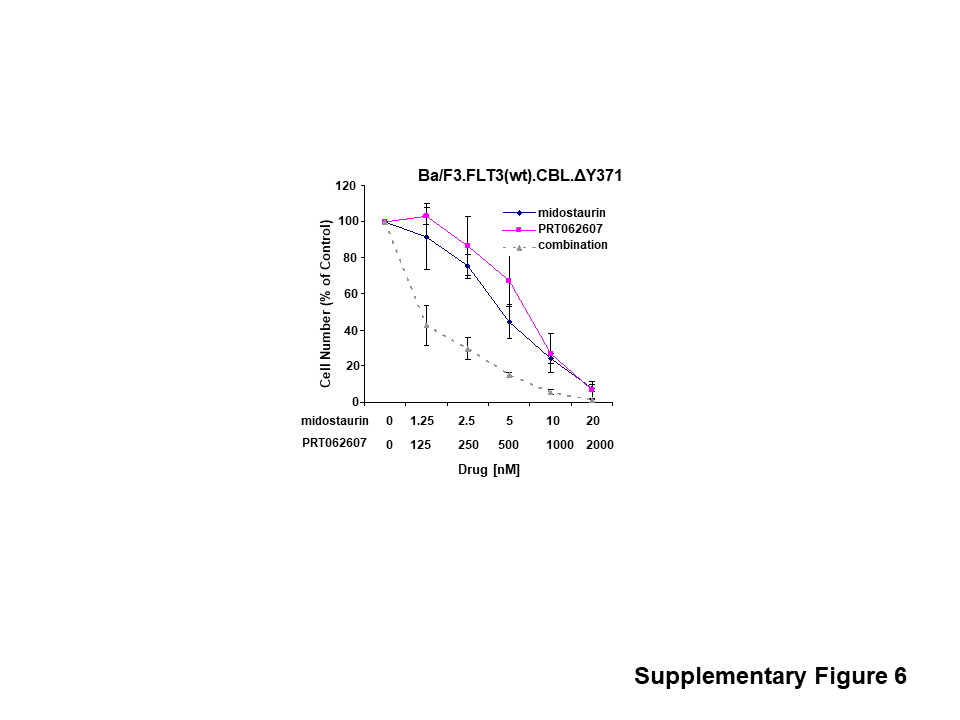

Supplement: Supplementary file 6 [file JCMM-24-2145-s006.TIF]

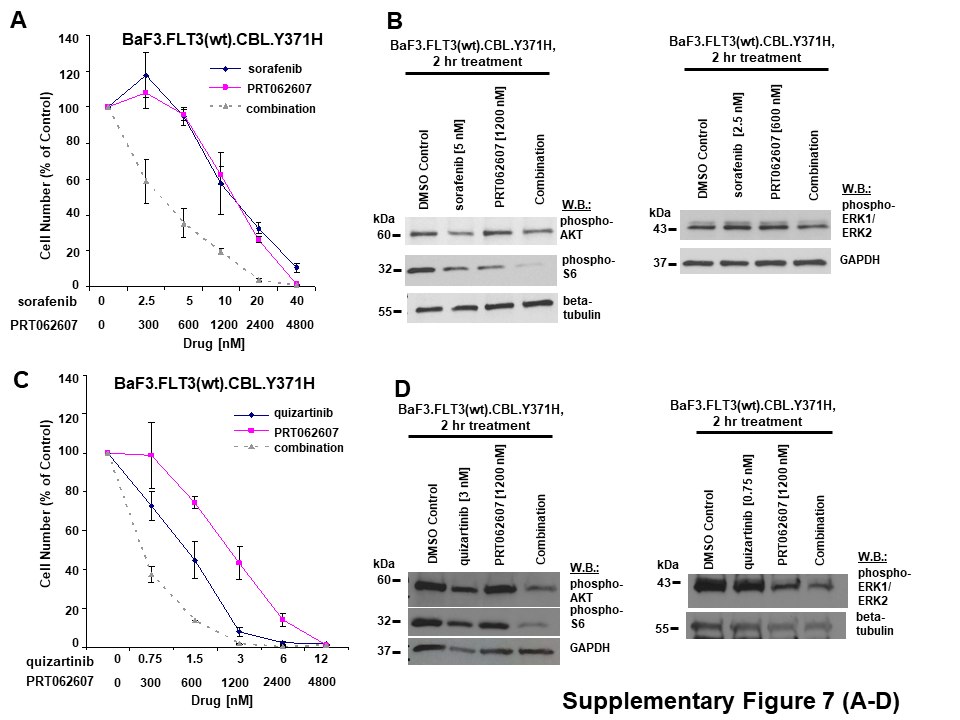

Supplement: Supplementary file 7 [file JCMM-24-2145-s007.TIF]

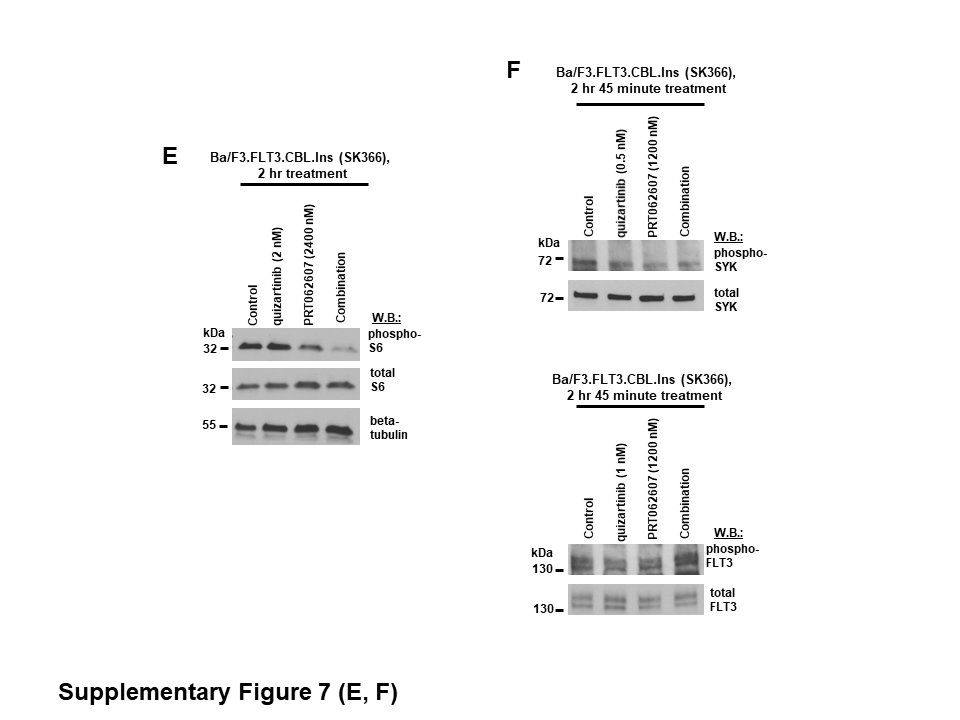

Supplement: Supplementary file 8 [file JCMM-24-2145-s008.TIF]

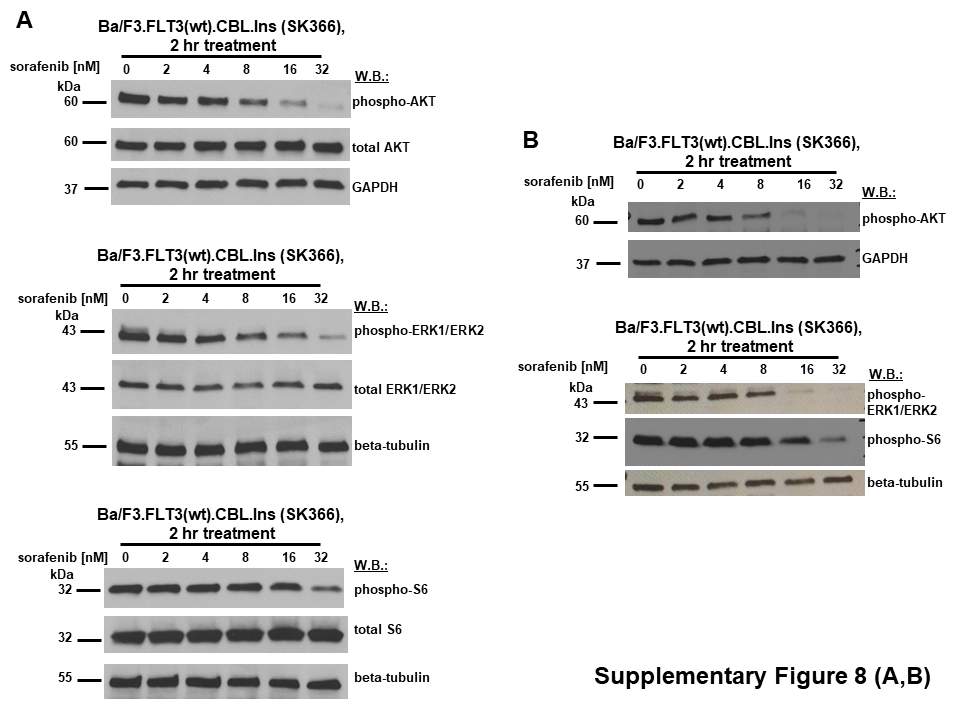

Supplement: Supplementary file 9 [file JCMM-24-2145-s009.TIF]

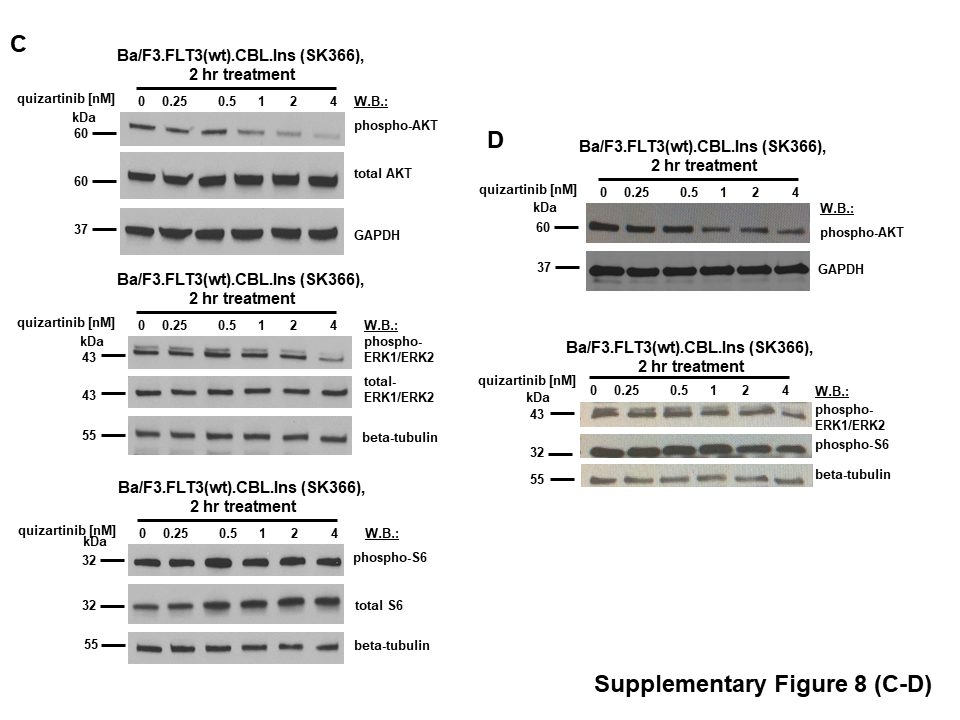

Supplement: Supplementary file 10 [file JCMM-24-2145-s010.TIF]

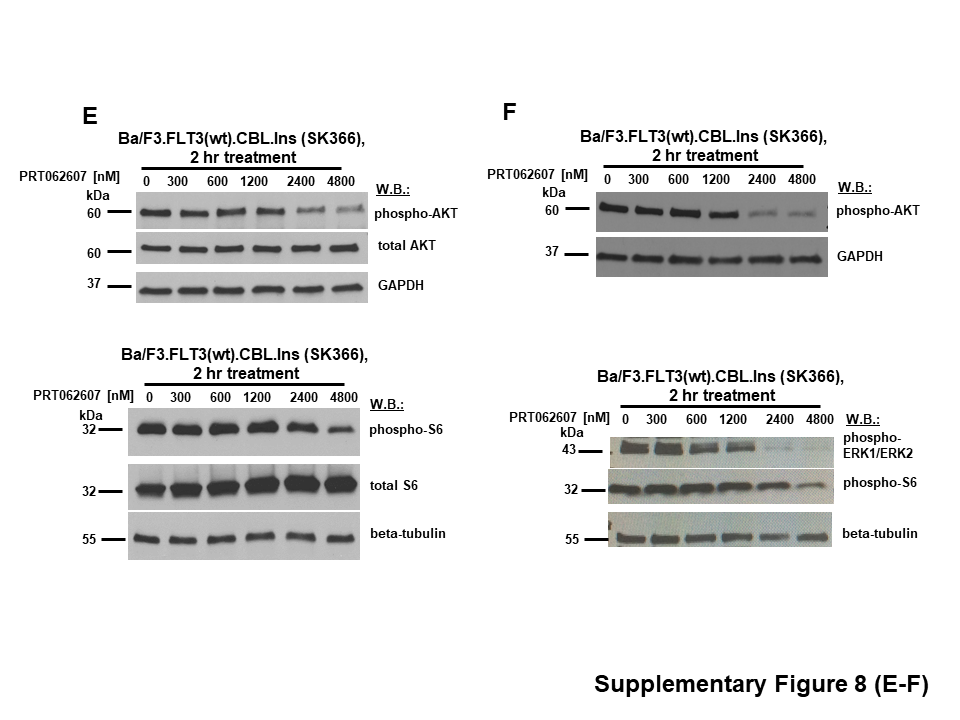

Supplement: Supplementary file 11 [file JCMM-24-2145-s011.TIF]

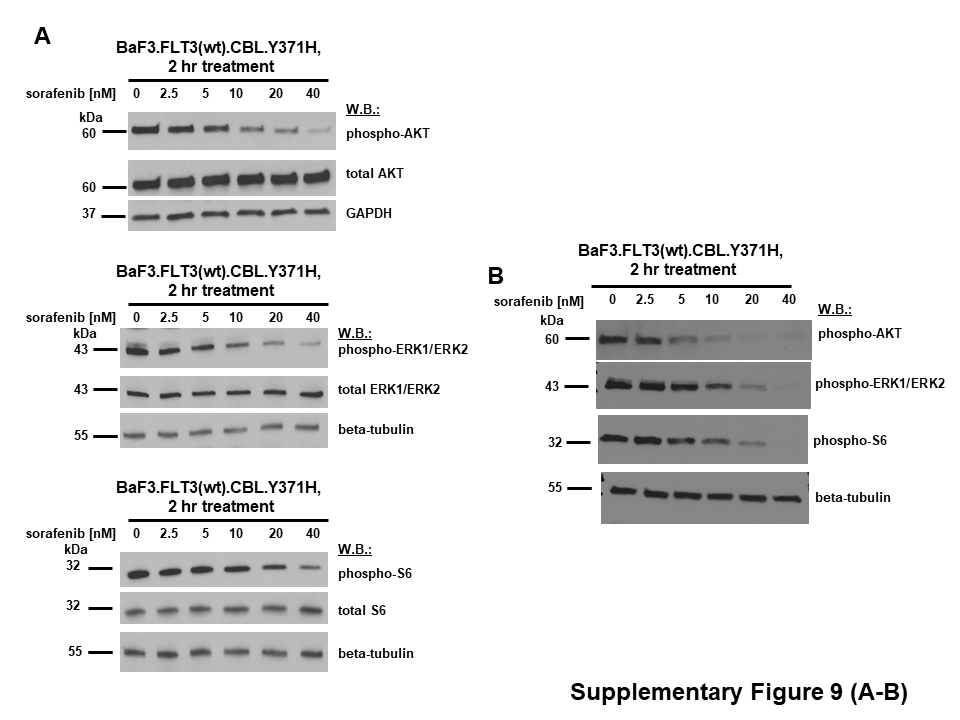

Supplement: Supplementary file 12 [file JCMM-24-2145-s012.TIF]

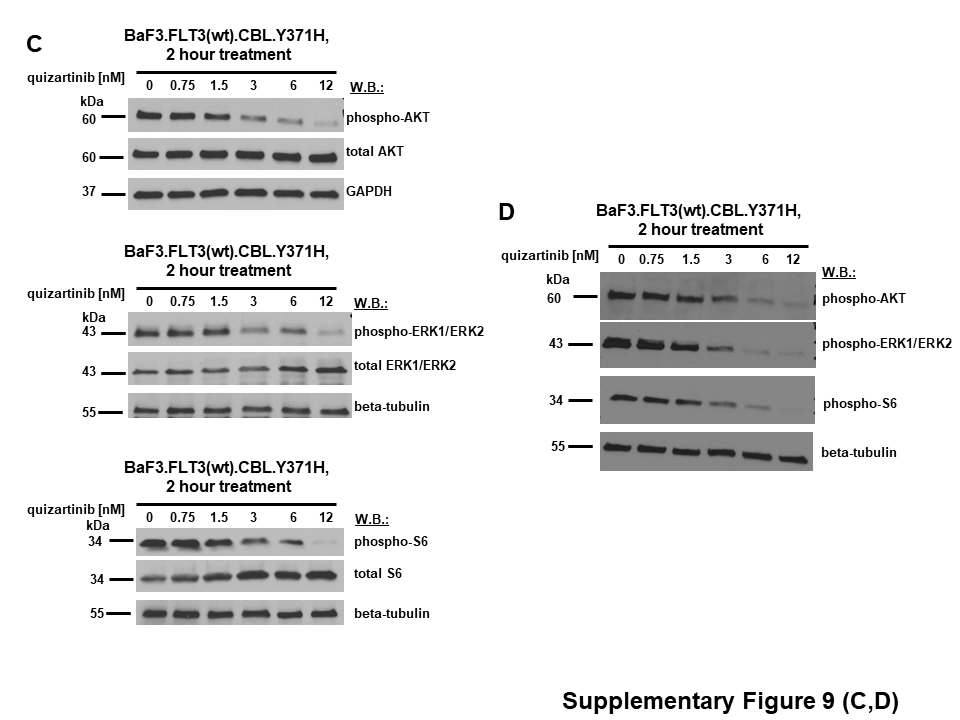

Supplement: Supplementary file 13 [file JCMM-24-2145-s013.TIF]

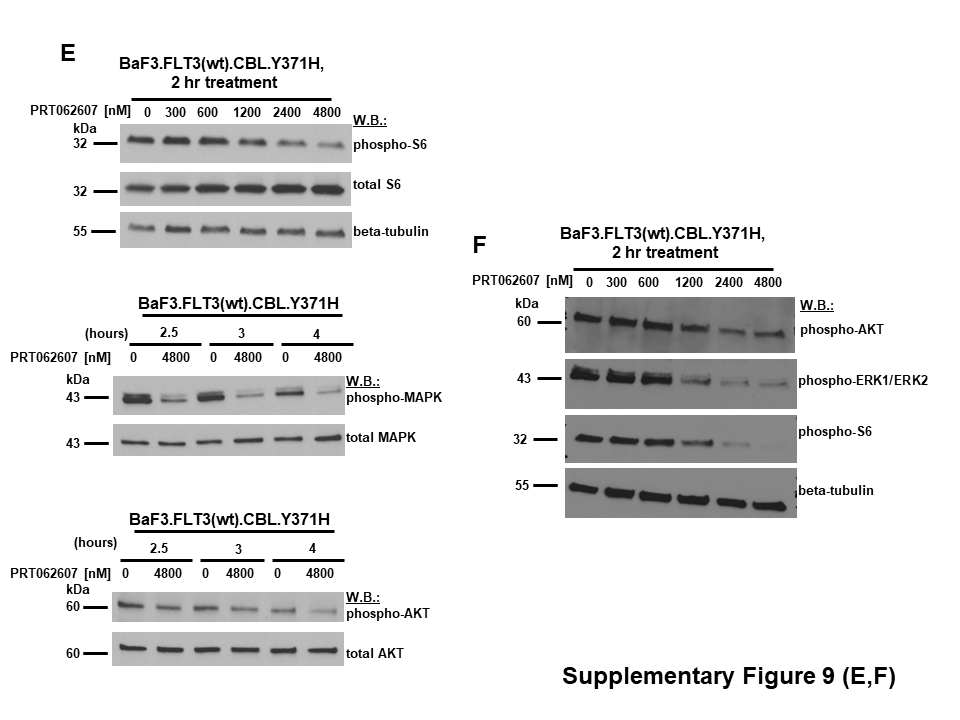

Supplement: Supplementary file 14 [file JCMM-24-2145-s014.TIF]

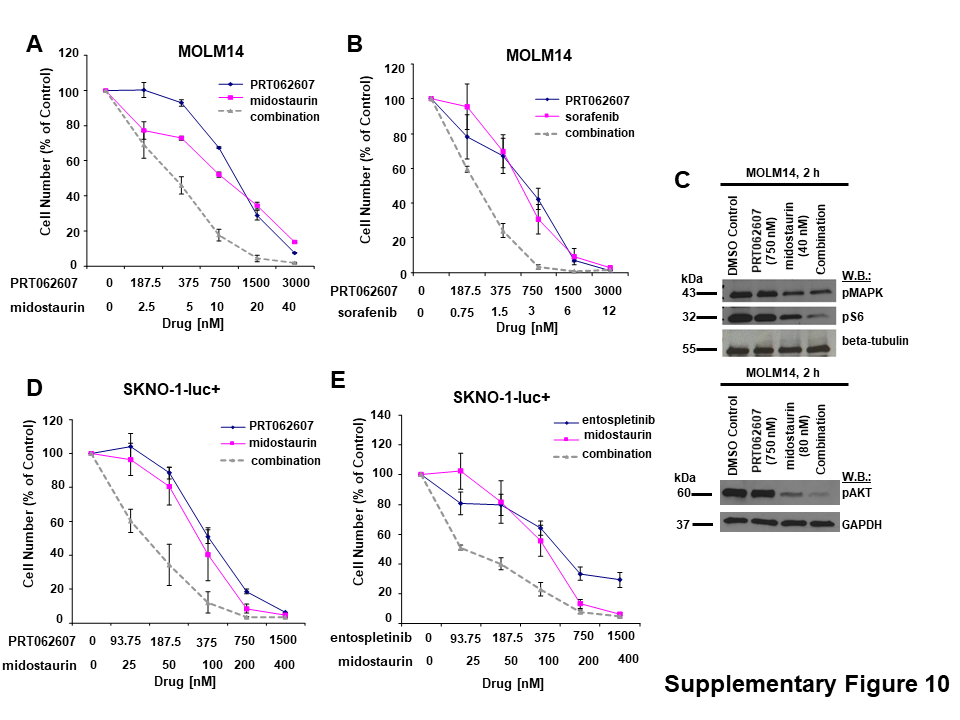

Supplement: Supplementary file 15 [file JCMM-24-2145-s015.TIF]

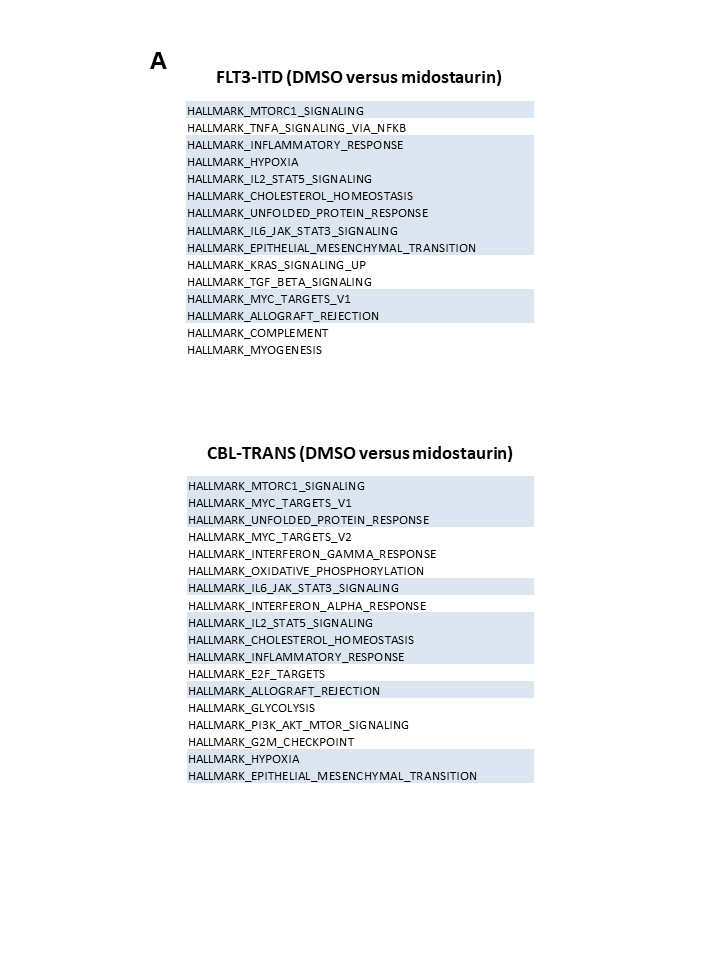

Supplement: Supplementary file 16 [file JCMM-24-2145-s016.TIF]

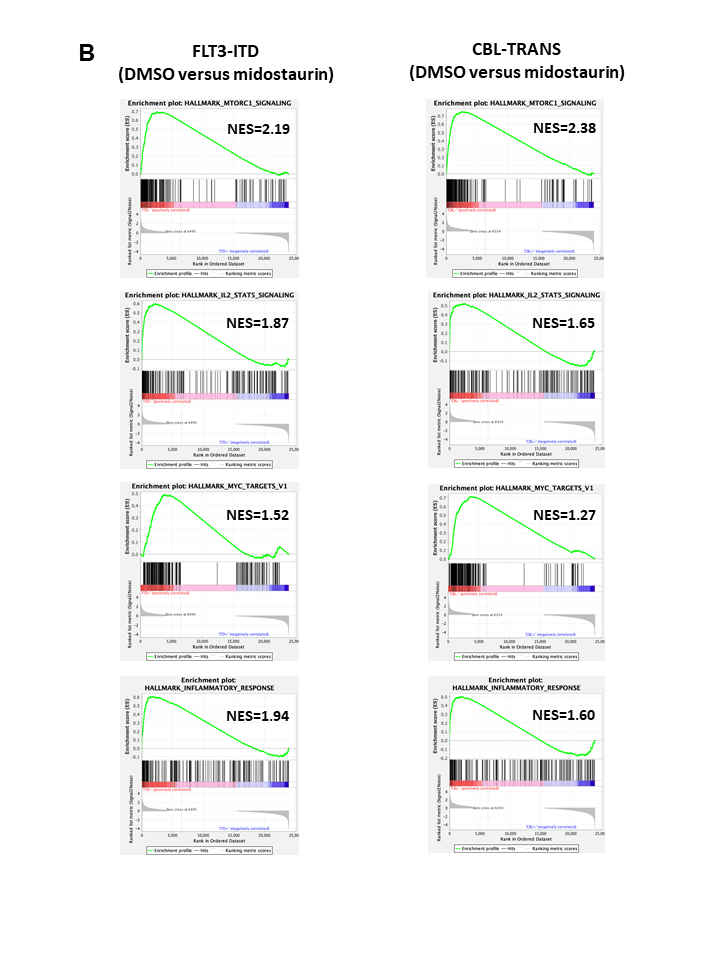

Supplement: Supplementary file 17 [file JCMM-24-2145-s017.TIF]
