# Supplementary material for: The combination of FLT3 and SYK kinase inhibitors is toxic to leukaemia cells with CBL mutations
Source: J Cell Mol Med. 2020 Jan 14;24(3):2145–56. doi: 10.1111/jcmm.14820 (PMC7011134; doi:10.1111/jcmm.14820)
Supplement: Supplementary file 18 [file JCMM-24-2145-s018.doc]

| **Patient** | **Diagnosis** | **Age** | **Gender** | **BM blast percent** | **Plt** | **Hct** | **WBC** | **Karyotype** | **Hgb** | **History of HSCT** | **CBL mutation/Other mutations** |
| --- | --- | --- | --- | --- | --- | --- | --- | --- | --- | --- | --- |
| Primary  mutant CBL  AML1 | New AML | 70 | F | 52 | 119 | 28.2 | 5.29 | Normal | 9.4 | Yes | CBL (D390V )  ASXL1  (E635fs*15)  RUNX1  (R166fs*47 )  RUNX1  (H242fs*14)  SRSF2  (P95R) |
| Primary mutant CBL  AML2 | AML  remission | 69 | M | 3 | 10 | 31.3 | 4.53 | 1 chromosomal abnormality | 11.2 | Yes | CBL (C416R)  IDH2 (R140Q)  SRSF2 (P95L)  STAG2 (W743*)  STAG2  (S174fs*) |
| Primary mutant CBL  CMML1 | CMML | 52 | M | <5* | 137 | 38.8 | 35.04 | Normal | 12.5 | No | CBL (I383M), CBL (C384Y)  AXL1  TET2 (Q1030)  TET2 (E1141fs*) |

| **Patient**  **Sample** | **Gender**  **Age** | **Clinical diagnosis/Clinical history** | **Blasts** | **Cytogenetics and genetic mutations** |
| --- | --- | --- | --- | --- |
| **Primary wt FLT3**  **AML2** | Male | Clinical diagnosis: AML in relapse  Clinical history: AML relapsed after transplant with refractory disease after 2 cycles on 14-222 MUC1+ decitabine | 95% (aspirate),  95%  (biopsy) | 45,XY,t(1;5)(q22;q33),t(3;4)(q27;q21),-7,inv(12)(p13q15),var(22)(p12)c[20].ish t(1;5)(PDGFRB-;PDGFRB+)[5]  Genetic mutations:  EZH2 p.R690H(37.5% of 432 reads)  PTPN11 p.N308D (28.5% of 695 reads)  RUNX1 p.N254fs (32% of 147 reads)  Read count analysis shows loss of IKZF1 and EGR (on 7p), loss of CUX1, LUC7L2, BRAF and EZH2 (on 7q), loss of ETV6 (on 12p).  FLT3-ITD is not detected. |
| **Primary FLT3-ITD**  **AML3** | Male | AML | 99% | 47,XY,+13[3]/46,XY[16]/92,XXYY[2]  FLT3-ITD detected: Insertion of 12 nt 3' to nt 1826, followed by a duplication of nt 1788 - 1826 (total = 51 bp). The ITD is 7% of the total flt3 alleles in the specimen; this normally corresponds to 14% blasts. However, the specimen had 93% blasts on the day of this analysis. Apparently, there is a minor clone with a 51bp ITD. |
| **Primary wt FLT3**  **AML5** | Male | Acute monoblastic leukemia, FAB M5a subtype | 90% | 47,XY,+8[3]/46,XY,-7,+8[17]  FLT3-ITD is not detected. |

Supplementary Table 1. Patient information.

*CMML is divided into two classifications based on cell counts in the blood and bone marrow. CMML-1 is characterized by blasts that make up less than 5% of white cells in blood and less than 10% of cells in bone marrow. CMML-2 is characterized by blasts that make up 5-20% of white cells in blood or 10-20% of cells in bone marrow.
