# Supplementary material for: The combination of FLT3 and SYK kinase inhibitors is toxic to leukaemia cells with CBL mutations
Source: J Cell Mol Med. 2020 Jan 14;24(3):2145–56. doi: 10.1111/jcmm.14820 (PMC7011134; doi:10.1111/jcmm.14820)
Supplement: Supplementary file 20 [file JCMM-24-2145-s020.doc]

Supplementary Materials and Methods

*Cell lines*

Human cell lines were authenticated within 6 months of manuscript preparation through cell line short tandem repeat (STR) profiling (DDC Medical, Fairfield, OH and Molecular Diagnostics Laboratory, Dana-Farber Cancer Institute, Boston, MA). All cell lines tested matched >80% with lines listed in the ATCC or DSMZ Cell Line Bank STR and were confirmed to be virus- and *Mycoplasma*-free.

*Chemical compounds and biologic reagents*

For *in vitro* studies, midostaurin, crenolanib, and quizartinib were purchased from Haoyuan chemexpress. Sorafenib was purchased from LC Laboratories. Gilteritinib (Hydrochloride) was purchased from Chemietek (Indianapolis, IN). Entospletinib and PRT062607 were purchased from Selleckchem (Houston, TX). All drugs were dissolved in DMSO to obtain a 10 mM stock solution. Serial dilutions were then made, to obtain final dilutions for cellular assays with a final concentration of DMSO not exceeding 0.1%.

*Drug combination studies*

For drug combination assays, we first quantified cells for seeding using the Trypan Blue exclusion assay. CellTiter-Glo was then carried out for proliferation studies. We added single agents simultaneously at fixed ratios to cells. Cell viability was ten expressed as a function of growth affected (FA) drug-treated versus DMSO control cells, and data were analyzed by Calcusyn software (Biosoft, Ferguson, MO and Cambridge, UK). This software was used for measurement of synergy or antagonism and is based on isobologram generation and the method of Chou and Talalay (1984) [23], which uses the median effect principle to quantify drug combination effects to assess whether or not they are greater than those expected from a simple addition of the single agent effects. Following determination of the ED_50_ or IC_50_ of each drug, combinations are analyzed where the concentrations are fractions or multiples of the ED/IC_50_. Statistic analysis is part of the computations, and combination indices or values generated by the Calcusyn software are either less than one (suggesting synergy) or greater than one (suggesting antagonism).

*AML patient cells*

All samples were obtained under approval of the Dana Farber Cancer Institute Institutional Review Board. Informed consent was obtained from all subjects, and the research was carried out according to the World Medical Association Declaration of Helsinki.

Mononuclear cells were isolated from peripheral blood or bone marrow samples from AML or CMML patients identified as harboring mutant CBL by density gradient centrifugation through Ficoll-Paque Plus (Amersham Pharmacia Biotech AB, Uppsala, Sweden) at 2000 rpm for 30 minutes, followed by two washes in 1X PBS.

*Cell proliferation studies*

The Trypan blue exclusion assay, previously described [13], was utilized for cell counting prior to seeding for CellTiter-Glo experiments. CellTiter-Glo (Promega, Madison, WI) was used for proliferation studies according to manufacturer instructions. Cell viability is shown in graphs as the percentage of control (untreated) cells; error bars represent the standard deviation for each data point.

.
