# Supplementary material for: The combination of FLT3 and SYK kinase inhibitors is toxic to leukaemia cells with CBL mutations
Source: J Cell Mol Med. 2020 Jan 14;24(3):2145–56. doi: 10.1111/jcmm.14820 (PMC7011134; doi:10.1111/jcmm.14820)
Supplement: Supplementary file 21 [file JCMM-24-2145-s021.doc]

Supplementary Figure Legends

**Supplementary Figure 1.** **Comparison of effects of targeted tyrosine kinase inhibitors Ba/F3 cells expressing wt human FLT3 alone or human CBL (ΔY371, Y371H, or insert (Ins (SK366)).** Proliferation assays were carried out. Cell lines were treated for 3 days. Shown as controls are growth factor-dependent parental Ba/F3, as well as Ba/F3 cells expressing wt human FLT3 (Ba/F3.FLT3(wt)) and Ba/F3 cells co-expressing wt human FLT3 and wt human CBL (Ba/F3.FLT3(wt).CBL(wt)). All three cell lines not expressing mutant CBL were cultured and tested in the presence of 20% WEHI-conditioned media, used as a source of IL-3. Cell lines were treated with midostaurin (A), sorafenib (B), PRT062607 (C), gilteritinib (D), crenolanib (E), and quizartinib (F).

**Supplementary Figure 2. Comparison of the potencies of PRT062607 and entospletinib against Ba/F3 cells co-expressing wt human FLT3 and mutant human CBL (Y371H or Ins (SK366)).** Proliferation assays were carried out. Inhibitors were tested in parallel for 3 days against BaF3.FLT3(wt).CBL.Y371H (A) or Ba/F3.FLT3(wt).CBL.Ins (SK366) (B).

**Supplementary Figure 3.** **Comparison of wt FLT3 and SYK expression and activity between Ba/F3-wt FLT3 and Ba/F3-wt FLT3+mut CBL** (A) IP of FLT3 in Ba/F3.FLT3(wt) cells (left panel) and Ba/F3.FLT3(wt).CBL.Ins (SK366) cells (right panel) followed by immunoblotting with pTYR. (B) FLT3 immunoblots of lysates from Ba/F3-wt FLT3 cells (cultured in the presence of WEHI as a source of IL-3) and Ba/F3 cells co-expressing wt FLT3 and mutant CBL. (C) FLT3 immunoblot of lysates from Ba/F3-wt FLT3 cells (cultured in absence of WEHI as a source of IL-3, overnight) and Ba/F3 cells co-expressing wt FLT3 and mutant CBL. (D) SYK immunoblot of lysates from Ba/F3-wt FLT3 cells (cultured in the absence of WEHI as a source of IL-3, overnight) and Ba/F3 cells co-expressing wt FLT3 and mutant CBL

**Supplementary Figure 4. Effects of midostaurin *in vivo* against mice harboring Ba/F3.FLT3(wt).CBL.Ins (SK366)-luc+ cells.** Effects of midostaurin *in vivo* against mice harboring Ba/F3.FLT3(wt).CBL.Ins (SK366)-luc+ cells. Supine and Prone (Low Scale), Day 1 Pre-treatment Representative Images (n=5)

**Supplementary Figure 5. Effects of midostaurin *in vivo* against mice harboring Ba/F3.FLT3(wt).CBL.Y371H-luc+ cells.** Effects of midostaurin *in vivo* against mice harboring Ba/F3.FLT3(wt).CBL.Y371H-luc+ cells. Supine and Prone (High Scale), Days 1-8. Shown are representative bioluminescence images (n=5).

**Supplementary Figure 6. Effects of midostaurin alone and in combination with PRT062607 against mutant CBL-expressing Ba/F3 cells.** Effects of midostaurin, alone and in combination with PRT062607, on proliferation of Ba/F3.FLT3(wt).CBL.ΔY371 cells. Cell lines were treated for 3 days.

**Supplementary Figure 7 (A-D). Effects of TKIs alone and in combination with PRT062607 against mutant CBL-expressing Ba/F3 cells.** (A,C) Effects of TKIs, alone and in combination with PRT062607 on proliferation of Ba/F3.FLT3(wt).CBL.Y371H cells. Cell lines were treated for 3 days. (B,D) Effects of sorafenib or quizartinib, alone and in combination with PRT062607, on the activity of signaling molecules downstream of FLT3 in Ba/F3 cells co-expressing human wt FLT3 and mutant CBL (Y371H)). Cells were treated for 2 hours.

**Supplementary Figure 7 (E-F). Effects of quizartinib alone and in combination with PRT062607 against mutant CBL-expressing Ba/F3 cells.** (E) Effects of quizartinib, alone and in combination with PRT062607, on the activity of S6 in Ba/F3.FLT3(wt).CBL.Ins (SK366) cells. Cells were treated for 2 hours. This immunoblot was performed independently of those shown in Figure 6. (F) Effects of quizartinib, alone and in combination with PRT062607, on the activity of SYK (upper panel) or FLT3 (lower panel) in Ba/F3.FLT3(wt).CBL.Ins (SK366) cells. Cells were treated for 2 hours and 45 minutes.

**Supplementary Figure 8 (A-B) Effects of sorafenib as a single agent on the activity of signaling molecules downstream of FLT3 in Ba/F3 cells co-expressing human wt FLT3 and mutant CBL.Ins (SK366).** Two individual immunoblotting experiments are shown for sorafenib-treated cells, both with (A) and without (B) total protein levels shown. Cells were treated for 2 hours prior to lysate preparation and immunoblotting.

**Supplementary Figure 8. (C-D) Effects of quizartinib as a single agent on the activity of signaling molecules downstream of FLT3 in Ba/F3 cells co-expressing human wt FLT3 and mutant CBL.Ins (SK366).** Two individual immunoblotting experiments are shown for quizartinib-treated cells, both with (C) and without (D) total protein levels shown. Cells were treated for 2 hours prior to lysate preparation and immunoblotting.

**Supplementary Figure 8 (E-F) Effects of PRT062607 as a single agent on the activity of signaling molecules downstream of FLT3 in Ba/F3 cells co-expressing human wt FLT3 and mutant CBL.Ins (SK366).** Two individual immunoblotting experiments are shown for PRT062607-treated cells, both with (E) and without (F) total protein levels shown. Cells were treated for 2 hours prior to lysate preparation and immunoblotting.

**Supplementary Figure 9. (A-B) Effects of sorafenib as a single agent on the activity of signaling molecules downstream of FLT3 in Ba/F3 cells co-expressing human wt FLT3 and mutant CBL.Y371H.** Two individual immunoblotting experiments are shown for sorafenib-treated cells, both with (A) and without (B) total protein levels shown. Cells were treated for 2 hours prior to lysate preparation and immunoblotting.

**Supplementary Figure 9. (C-D) Effects of quizartinib as a single agent on the activity of signaling molecules downstream of FLT3 in Ba/F3 cells co-expressing human wt FLT3 and mutant CBL.Y371H.** Two individual immunoblotting experiments are shown for quizartinib-treated cells, both with (C) and without (D) total protein levels shown. Cells were treated for 2 hours prior to lysate preparation and immunoblotting.

**Supplementary Figure 9. (E-F) Effects of PRT062607 as a single agent on the activity of signaling molecules downstream of FLT3 in Ba/F3 cells co-expressing human wt FLT3 and mutant CBL.Y371H.** Two individual immunoblotting experiments are shown for PRT062607-treated cells, both with (E) and without (F) total protein levels shown. Cells were treated for the indicated times prior to lysate preparation and immunoblotting.

**Supplementary Figure 10. Effects of FLT3 inhibitors alone and combined with PRT062607 or entospletinib against FLT3-ITD/mutant CBL-expressing MOLM14 and wt FLT3/wt CBL-expressing SKNO-1-luc+ cells.** Cell lines were treated for approximately 3 days. PRT062607 was tested in a proliferation assay in combination with midostaurin (A) or sorafenib (B) against MOLM14 cells, and midostaurin was tested in combination with PRT062607 (D) or entospletinib (E) against SKNO-1-luc+ cells. (C) Investigation of effects of midostaurin, alone and combined with PRT062607, on signaling molecules downstream of FLT3 in MOLM14 cells.

**Supplementary Figure 11. RNAseq analysis.** (A) Significantly changed pathways (nominal p-value <0.05; q-value <0.05). Common changes are highlighted. Selected enrichment plots. The normalized enrichment scores (NES) are indicated.
